# Adipose-Derived Circulating Exosomes Promote Protection of the Pulmonary Endothelial Barrier by Inhibiting EndMT and Oxidative Stress through Down-Regulation of the TGF-*β* Pathway: A Potential Explanation for the Obesity Paradox in ARDS

**DOI:** 10.1155/2022/5475832

**Published:** 2022-05-05

**Authors:** Di Qi, Wang Deng, Xiaorui Chen, Shulei Fan, Junnan Peng, Xumao Tang, Daoxin Wang, Qian Yu

**Affiliations:** Department of Respiratory Medicine, Second Affiliated Hospital of Chongqing Medical University, Chongqing, China

## Abstract

The “obesity paradox in acute respiratory distress syndrome” (ARDS) refers to the phenomenon in which obesity is associated with higher morbidity but lower mortality in patients with ARDS. Endothelial-to-mesenchymal transition (EndMT) represents a key link in the interaction between endothelial disruption and mesenchymal fibrosis under inflammatory and oxidative conditions, which represent the intersectional pathophysiology of ARDS. Adipose tissue is considered to constitute the major source of circulating exosomal microRNAs (miRNAs), which act as genetic forms of adipokines for cell–cell crosstalk. We aimed to demonstrate the regulation and mechanism of adipose-derived exosomes in the obesity paradox in ARDS. High-fat-induced obese mice and lean control mice were subjected to ARDS insult to investigate the effects of obesity on ARDS and microarray analysis was performed to screen for differences in circulating miRNAs. In addition, mice and pulmonary endothelial cells were administered with adipose-derived exosomal miR-122-5p to investigate the underlying molecular mechanisms. We found high-fat diet-induced obesity protected against ARDS in mice by reinforcing endothelial barrier and attenuating fibroproliferation. Circulating exosomes produced in the obese state mediated these protective effects by inhibiting EndMT and oxidative stress. Mechanistically, adipose-derived exosomal miR-122-5p promoted the integrity and function of pulmonary endothelial barrier and alleviated fibrogenesis by suppressing EndMT and oxidative stress through down-regulation of the transforming growth factor *β*1 (TGF-*β*1)/TGF-*β* receptor 1 (TGF-*β*R1)/Smad2 pathway *in vivo* and *in vitro.* In conclusion, adipose-derived circulating exosomal miR-122-5p protects against ARDS by reinforcing pulmonary endothelial barrier through inhibition of EndMT and oxidative stress via down-regulation of the TGF-*β* pathway, which propose a potential explanation for the obesity paradox in ARDS and indicate promising prospects for adipose-derived exosomes in cell-free therapies for ARDS.

## 1. Introduction

Acute respiratory distress syndrome (ARDS), a severe form of acute lung injury, is a relatively common and lethal syndrome [1]. According to an international study, ARDS occurred in 10.4% of all patients admitted to the intensive care unit, with 46% mortality in patients with severe ARDS [2]. Currently, no effective pharmacologic therapies exist for ARDS. Disruption of the pulmonary microvascular endothelial barrier plays a key role in ARDS pathophysiology. Pulmonary fibrosis can occur even during the acute phase of ARDS, which may further aggravate microvascular dysfunction [3]. Endothelial-to-mesenchymal transition (EndMT) is a cellular phenotype differentiation process whereby endothelial cells lose their endothelial properties and acquire mesenchymal-like cellular features, thus acting as a key link in the interaction between endothelial disruption and mesenchymal fibrosis under inflammatory and oxidative conditions. A number of mediators, including inflammatory cytokines, growth factors, and reactive oxygen species (ROS), induce the progression of EndMT [4–6]. Therapies aimed at mitigating EndMT are supposed to restore endothelial barrier integrity and alleviate pulmonary fibrosis, thereby improving the clinical outcomes of patients with ARDS.

The “obesity paradox in ARDS” refers to the phenomenon in which obesity is associated with higher morbidity but lower mortality in patients with ARDS [7, 8]. In the general population, obesity and being overweight are associated with an increased risk of death, but a decrease in mortality has been documented in specific critical diseases such as ARDS [9–12]. Animal studies also support the protective capacity of obesity against lung injury [13]. Although adipose tissue is an important endocrine tissue that contributes to the progression of ARDS, few studies have focused on adipose–lung crosstalk, and the role of adipose tissue-derived exosomal miRNAs in the pathogenesis of ARDS remains largely unexplored. Recently, we reported a new mechanism whereby high-fat diet (HFD)-induced obesity protected against ventilator-induced lung injury (VILI) by regulating pulmonary endothelial barrier homeostasis via adipose-derived exosomes [14]. However, rigorous studies on the molecular mechanism are still needed to clarify the impact of obesity on ARDS.

MicroRNAs (miRNAs) are small noncoding RNAs that regulate gene expression and biological processes [15]. A large proportion of miRNAs existing in exosomes can be taken up by recipient cells to subsequently modulate cellular functions, thus serving as important mediators of intercellular communication. As an important endocrine tissue, adipose tissue secretes various adipokines involved in the progression of acute lung injury [16–18]. Recently, an innovative study documented that adipose tissue constitutes a major source of circulating exosomal miRNAs, and these miRNAs can regulate gene expression in distant tissues thereby serving as genetic forms of adipokines for cell–cell crosstalk [19–22].

Hence, in the present study, we hypothesized that adipose-derived exosomal miRNAs mediate the protective ability of obesity against ARDS by regulating pulmonary endothelial barrier homeostasis. First, we explored the effects of obesity on ARDS *in vivo*; subsequently, we investigated the role of adipose-derived exosomes in pulmonary endothelial barrier under ARDS *in vivo* and *in vitro*. Furthermore, we analyzed the differences in circulating exosomal miRNAs between obese and lean mice with ARDS and finally elucidated the underlying molecular mechanisms of the effects.

## 2. Materials and Methods

### 2.1. Mouse Model of ARDS and Exosome Treatment

From 5 weeks of age, male wild-type C57BL/6J mice of SPF grade were given free access to purified high-fat diet (HFD; 40 kcal% fat; Gempharmatech Co., Ltd., China) or normal chow diet (NCD; 10 kcal% fat) for 12 weeks. Animal procedures were approved by the ethics committee of Chongqing Medical University and implemented according to the instructions of the National Institutes of Health Guide for the Care and Use of Laboratory Animals. Mice were anesthetized with sodium pentobarbital (50 mg/kg administered intraperitoneally), after which the trachea and right internal jugular vein were exposed. ARDS mouse models were established with intratracheal instillation of 5 mg/kg lipopolysaccharide (LPS; *Escherichia coli* O111:B4; Sigma-Aldrich, St. Louis, MO 63103, USA) in 50 *μ*L of sterile phosphate-buffered saline (PBS) via an 18-G catheter. Circulating exosomes in obese mice are reported to be present at approximately 30 *μ*g per mouse [22, 23]. After experimental optimization, we administered 30 *μ*g of exosomes from the serum of obese mice (100 *μ*g/mL in 300 *μ*L of PBS every week for 3 weeks) or a PBS control to lean recipient mice via tail vein injection before ARDS was established. In some experiments, recombinant mouse transforming growth factor *β*1 (TGF-*β*1) peptide (10 *μ*g/mL in a total volume of 100 *μ*L; R&D Systems Inc; Minneapolis, MN, USA) with or without intraperitoneal administration of the TGF-*β*1 receptor I (TGF-*β*RI) inhibitor SB-431542 (10 mg/kg; Sigma-Aldrich, St. Louis, MO 63103, USA) was administered to mice one hour before LPS instillation. After 7 days of the LPS insult, mice were sedated, anesthetized, and humanely subjected to cervical dislocation for euthanasia according to the Interdisciplinary Principles and Institutional Animal Care and Use Committee guidelines.

### 2.2. Exosome Isolation and Identification *In Vivo* and *In Vitro*

Firstly, extraction of serum exosomes from mice was performed using total exosome isolation (from serum) reagent (Invitrogen, California, USA). Serum sample was centrifuged at 2,000×*g* for 30 minutes to remove cell debris. Subsequently, the serum was transferred to a new tube and mixed with 0.2 volumes of total exosome isolation reagent. The mixture was incubated at 4°C for 30 minutes and centrifuged at 10,000×*g* for 15 minutes. Finally, the exosomes were suspended in PBS, centrifuged, washed, and resuspended in 100 *μ*L of PBS for downstream analysis. Next, for isolation of exosomes from adipose tissue, visceral adipose tissue (VAT) was minced to 2 mm and precultured in Dulbecco's modified Eagle's medium (DMEM) containing 50 *μ*g/mL of gentamicin and 10% exosome-free fetal bovine serum (FBS; ScienCell, Carlsbad, CA, USA) for 1 hour. The tissue was then washed and centrifuged with DMEM before being cultured in DMEM for 24 hours. Exosomes were concentrated and collected by differential ultracentrifugation using centrifuge filters as previously described [14, 19, 24]. Finally, for isolation of adipocyte-derived exosomes, debris and dead cells were removed from the medium using centrifugation at 2,000×*g* for 10 minutes and the medium was then filtered through a 0.2 *μ*m filter. The filtered medium was ultracentrifuged at 100,000×*g* for 4 hours to collect exosomes. After washing with PBS, the exosome-containing pellets were resuspended in 100 *μ*L of PBS for further experiments. Transmission electron microscopy (TEM; HITACHI HT7700 TEM, Hitachi, Ltd., Tokyo, Japan) was used for observations and image analysis. For nanoparticle tracking analysis (NTA), an NTA system from Malvern (Nano ZS ZEN3600; Malvern Panalytical Ltd., Worcestershire, UK) was used to measure the size distribution of exosomes. Western blots of exosome protein markers were probed using antibodies against CD63 and TSG 101 (both 1 : 1,000; Proteintech Group Inc., Rosemont, USA).

### 2.3. miRNA Profiling Microarray

Exosomal miRNAs from serum were isolated using the exoRNeasy Serum/Plasma Maxi Kit (QIAGEN, Hilden, Germany). The miRNA expression profiles of serum exosomal miRNAs in lean and obese mice with ARDS were comparatively analyzed using a microarray (for all mature miRNAs of Mus musculus available in the 21.0 version of the miRBase database) by LC Sciences (Houston, TX, USA). Total RNA (5 *μ*g) was extended using poly(A) polymerase and ligated to an oligonucleotide tag for fluorescent staining. Microarray hybridization was conducted on *μ*Paraflo microfluidics chip. Cy3 dye was circulated through the chip, and fluorescent images were visualized using GenePix 4000B scanner (Molecular Devices, San Jose, CA, USA) with Array-Pro software (Media Cybernetics, Rockville, MD, USA) used for digital transformation. Values were calculated after background subtraction and normalization using a locally weighted regression. Values from triplicate samples in two groups were bioinformatically analyzed using two-tailed Student's *t* test by the OmicStudio tools (LC Sciences, Houston, TX, USA) to select the differential expression of miRNAs. Differences in miRNA expression were regarded as significant at *P* < 0.05. The predicted genes targeted to differentially expressed miRNAs were classified in accordance with the Kyoto Encyclopedia of Genes and Genomes (KEGG) pathway database to annotate the associated pathways.

### 2.4. Transfection of miR-122-5p Agomir or miR-122-5p Antagomir *In Vivo*

VAT of lean control mice was transfected with 5-carboxyfluorescein (5-FAM)-labeled or nonlabeled miR-122-5p agomir (10 nmol) or miR-122-5p antagomir (50 nmol) in 100 *μ*L RNase-free distilled water, as well as with their respective negative controls (NCs), every week for 3 weeks.

### 2.5. Exosome Labeling and Uptake *In Vivo* and *In Vitro*

PKH26 red fluorescent dye (Sigma-Aldrich) was used to label exosomes for 30 minutes at 37°C according to the manufacturer's instructions. The labeled exosomes were then ultracentrifuged at 100,000×*g* at 4°C for 1 hour, and washed in PBS three times. PKH26-labeled exosomes derived from adipose tissue of obese mice were intravenously administered to lean control mice 1 hour before the ARDS challenge. Lung tissues were isolated and fixed for immunofluorescent staining. *In vitro*, PKH26-labled exosomes derived from the concentrated medium of adipocytes were cocultured with human pulmonary microvascular endothelial cells (HPMECs) for 6 hours. Immunofluorescence analysis was conducted in lung tissues and HPMECs to detect exosome uptake. Cell nuclei were stained with dihydrochloride (DAPI) blue fluorescent dye (Cell Signaling Technology, Inc., Danvers, USA), and endothelial cell marker CD31 (1 : 2,000, Cell Signaling Technology) was detected using green fluorescence (Alexa Fluor 488 conjugated IgG; Abcam, Cambridge, MA, USA). Images were captured using a fluorescence microscope (TE2000-U; Nikon, Tokyo, Japan).

### 2.6. Lung Histology Evaluation

Mice were sedated and sacrificed according to the Institutional Animal Care and Use Committee guidelines. The aorta was cut to allow blood to drain from the lungs, and the trachea was exposed and inflated with 4% paraformaldehyde at constant pressure. The left lungs were excised, embedded in paraffin, and processed into 5 *μ*m sections for hematoxylin and eosin (H&E) and Masson's trichrome staining.

### 2.7. Lung Wet/Dry Weight Ratio

After the experiment, right lung tissue was harvested, weighed, and dried in an oven at 55°C for 72 hours. The dry weight was then measured, and the wet/dry (W/D) ratio was calculated.

### 2.8. Analysis of Bronchoalveolar Lavage Fluid (BALF)

Bronchoalveolar lavage was conducted by infusing 1 mL of sterile normal saline into the lungs through a trimmed 18-G catheter inserted into the trachea, and bronchoalveolar lavage fluid (BALF) was aspirated three times and centrifuged at 500×*g* for 20 minutes at 4°C. Bicinchoninic acid protein assay (BCA) Kit (Solarbio Technology Co., Ltd., Beijing, China) was used to detect the protein concentrations in the BALF supernatants.

### 2.9. Evans Blue-Dyed Albumin (EBDA) Concentrations in Lung Tissue

Pulmonary capillary permeability was evaluated by measuring EBDA concentrations. The right internal jugular vein of the mice was injected with EBDA (30 mg/kg; Invitrogen). Then, the mice were euthanized, and their lung tissues were harvested, weighed, and homogenized in 1 mL of PBS. Then, tissues were extracted in 2 mL of formamide for 24 hours at 60°C and centrifuged at 5,000×*g* for 30 minutes. The absorbance of the supernatants was measured using a microbiology reader (Bio-Tek Synergy HT; BioTek Instruments, Inc., Winooski, VT, USA), plotted against a standard curve, normalized, and converted to *μ*g EBDA/g lung tissue.

### 2.10. Collagen Quantification Assay

To measure collagen content in the lungs, a collagen quantification assay (Sigma-Aldrich) was performed. Briefly, lung tissue (100 mg) was dissociated in 1 mL of acetic acid, and sonicated on ice for 10 cycles. Samples were incubated at 4°C overnight and centrifuged at 10000×*g* for 15 minutes. An equal volume of NaOH was added to the supernatant to neutralize the sample. Samples and digest enzyme working solution were added to each well, and incubated at 37°C for 60 minutes. The working solution and development working solution were successively added after incubation for 15 minutes at 37°C. Fluorescence intensity was measured using the aforementioned Bio-Tek Synergy HT microplate reader at Ex/Em = 375/465 nm.

### 2.11. Enzyme-Linked Immunosorbent Assay (ELISA)

The levels of tumor necrosis factor *α* (TNF-*α*), interleukin (IL)-6, total matrix metalloproteinase 9 (MMP9), and TGF-*β* were determined using commercially available ELISA kits (R&D Systems) according to manufacturer's instructions.

### 2.12. HPMECs Culture

HPMECs were cultured in endothelial cell medium (ECM; ScienCell, Carlsbad, CA, USA) with 10% FBS, 1% endothelial cell growth supplement, and 1% penicillin/streptomycin (ScienCell). Cells were incubated at 37°C in 5% CO_2_ and starved without serum and growth supplement for 6 hours before each treatment. In some experiments, HPMECs were treated with LPS (100 ng/mL) in the absence or presence of recombinant human TGF-*β*1 protein (5 ng/mL for 6 hours, R&D Systems) for 72 hours. For neutralizing assay, HPMECs were preincubated with 1.25 *μ*g/mL of neutralizing antibody (R&D Systems) for 6 hours to block TGF-*β*1 signaling.

### 2.13. Differentiation of Preadipocytes into Adipocytes

A previous study suggested that the majority of exosomes released from adipose tissue are adipocyte derived [24]. Differentiation of human visceral preadipocytes (HPA-v; MeisenCTCC, China) was used to generate mature adipocyte cells. Preadipocyte growth supplement (5 mL), FBS (25 mL), and penicillin/streptomycin solution (5 mL) were added to 500 mL of preadipocyte medium (PAM) to prepare the complete medium for optimal growth of HPA-v cells. HPA-v cells were seeded in a T-75 flask at a density of 5 × 10^3^ cells/cm^2^ and expanded in PAM until confluency reached to 100%. To initiate mature adipocyte differentiation, PAM was removed and preadipocyte differentiation medium (PADM) was added. The medium was replaced with fresh PADM every 2–3 days, and the PADM was changed to adipocyte medium after 7–12 days. Mature adipocytes were maintained in adipocyte medium for up to 7 days for further experiments.

### 2.14. Transfection of miRNA Mimic and Inhibitor *In Vitro*

Adipocytes were transfected with 5-FAM-labeled or nonlabeled miR-122-5p mimic (50 nM) or inhibitor (100 nM), as well as the respective NCs for 48 hours using a riboFECT CP Transfection Kit (Guangzhou RiboBio Co., Ltd., Guangzhou, China) according to the manufacturer's instructions.

### 2.15. Luciferase Assays

**Figure 1:**
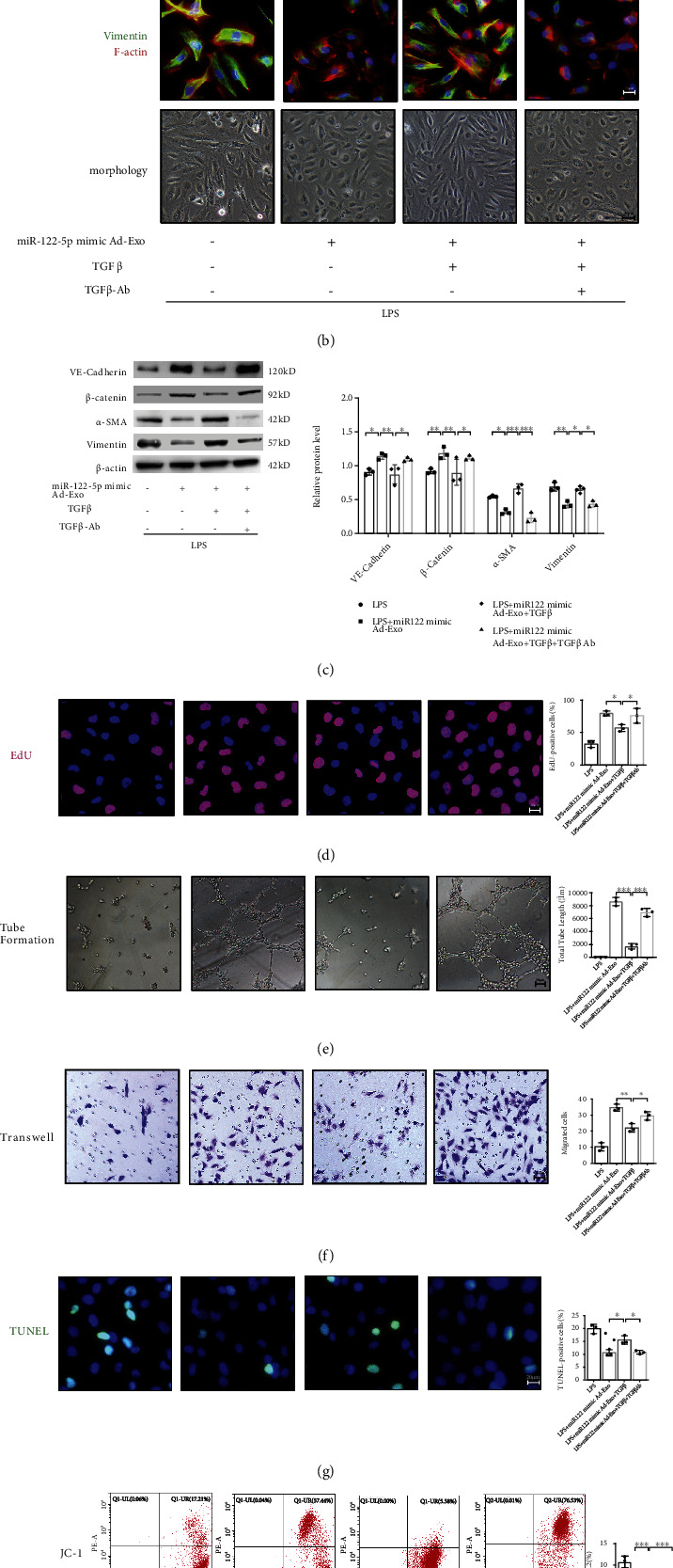
Adipocyte-derived exosomal miR-122-5p reinforces the pulmonary endothelial barrier by inhibiting EndMT and oxidative stress through down-regulating the TGF-*β*1/TGF-*β*R1/Smad2 pathway in HPMECs. (a) Dual-Luciferase reporter assay showed that human TGF-*β*1 is a direct target of miR-122-5p through the binding site in its 5′-UTR. The firefly luciferase activity of the reporter containing the 5′UTR-WT of human TGF-*β*1 was decreased to 62.61% by miR-122-5p, which was blocked by the reporter containing the 5′-UTR -MUT of human TGF-*β*1 (C787G, C797T, C807T). (b) Immunofluorescence staining, phalloidin F-actin staining, and cell morphology of HPMECs showed that the inhibitory effects of exosomal miR-122-5p on EndMT in LPS-insulted HPMECs were blocked by TGF-*β*1 treatment, but TGF*β*1-Ab overturned these effects. (c) Western blot analysis of the protein expression of VE-cadherin, *β*-catenin, *α*-SMA, and Vimentin in HPMECs. Relative abundances of protein bands were normalized to *β*-actin, as shown in the bar graphs. EdU staining (d), tube formation (e), transwell (f), TUNEL staining (g), and JC-1 staining (h) showed that the angiogenic and antioxidant capacity of exosomal miR-122-5p was abrogated by TGF-*β*1 treatment (5 ng/mL for 6 hours), but TGF-*β*1-Ab (1.25 *μ*g/ml for 6 hours) partially restored these capacities. (i) Western blot analysis of the protein expression of TGF-*β*1, and the phosphorylation of TGF-*β*R1 (p-TGF-*β*R1) and Smad2 (p-Smad2) in HPMECs. Relative abundances of protein bands were normalized to *β*-actin as shown in the bar graphs. Relative phosphorylation levels of protein are expressed normalized to the corresponding total protein. Representative images are shown from three replicated independent experiments. *n* = 6 per group (a). *n* = 3 per group for other groups. Data are presented as mean ± S.D. Significant differences are shown by ∗*P* < 0.05, ∗∗*P* < 0.01, and ∗∗∗*P* < 0.001.

**Figure 2:**
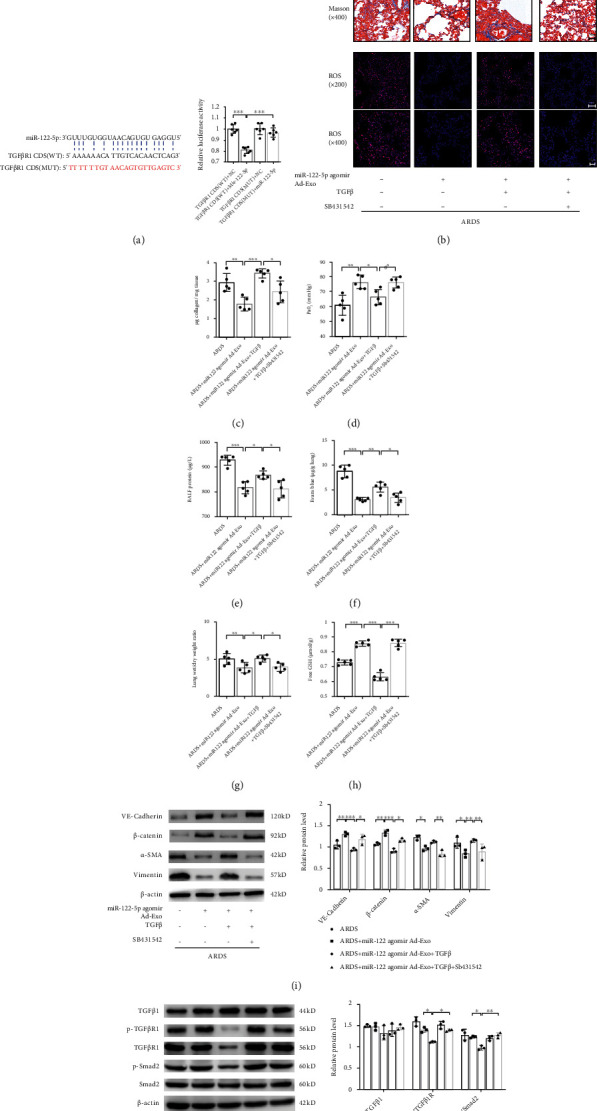
Adipose-derived exosomal miR-122-5p promotes the pulmonary endothelial barrier by inhibiting EndMT and oxidative stress through down-regulating the TGF-*β*1/TGF-*β*R1/Smad2 pathway in mice with ARDS. (a) Dual-Luciferase reporter assay showed that the CDS of mouse TGF-*β*R1 is a direct target of miR-122-5p. The firefly luciferase activity of the reporter containing the CDS-WT of mouse TGF-*β*R1 was decreased to 80.62% by miR-122-5p, which was blocked by the reporter containing the CDS-MUT of TGF-*β*R1 (complementary mutation). (b) Alterations of lung tissue by H&E, Masson's trichrome staining, and ROS production showed that the recombinant mouse TGF-*β*1 (10 *μ*g/mL) partially abolished the inhibitory effects of adipose-derived exosomal miR-122-5p on EndMT, whereas pretreatment with SB431542 (10 mg/kg) restored these favorable effects in mouse model of ARDS. The collagen content (c) PaO_2_ (d), BALF protein (e), EBDA extravasation (f), wet/dry ratio (g), and free GSH concentration (h) in mice with ARDS treated with adipose-derived exosomal miR-122-5p in the presence or absence of recombinant mouse TGF-*β*1 and SB431542. (i) Western blot analysis of the protein expression of VE-cadherin, *β*-catenin, *α*-SMA, and Vimentin in lungs. Relative abundances of protein bands were normalized to *β*-actin as shown in the bar graphs. (j) Western blot analysis of the protein expression of TGF-*β*1, and the level of p-TGF-*β*R1 and p-Smad2 in lungs. Relative abundances of protein bands were normalized to *β*-actin as shown in the bar graphs. Relative phosphorylation levels of protein are expressed normalized to the corresponding total protein. Representative images are shown from three replicated independent experiments. *n* = 6 per group (a). *n* = 3 mice per group ((b), (i), (j)). *n* = 5 mice per group (c)–(h). Data are presented as mean ± S.D. Significant differences are shown by ∗*P* < 0.05, ∗∗*P* < 0.01, and ∗∗∗*P* < 0.001.

Firefly and *Renilla* luciferase assays were performed to identify putative miRNA binding sites. As shown in Figures 1(a) and 2(a), the 5′-untranslated region (UTR) or mutated 5′-UTR (C787G, C797T, and C807T) of human TGF-*β* and coding sequences (CDS) or mutated CDS (complementary mutation) of mouse TGF-*β*R1 were inserted into a pMIR-REPORT miRNA expression reporter vector (Ambion, Austin, TX, USA) by OBiO Technology Corp., Ltd. (Shanghai, China). HPMECs were cotransfected in Opti-MEM (Gibco, Carlsbad, CA, USA) with 0.2 *μ*g of the firefly luciferase reporter vector and 0.01 *μ*g of the *Renilla* luciferase normalization control vector (pRL-CM; Promega, Madison, WI, USA), as well as with 100 nM miR-122-5p mimics or miRNA-NC using Lipofectamine 2000 (Invitrogen, California, USA). Cells were lysed, and firefly and *Renilla* luciferase activities were measured 48 hours post-transfection using the Dual-Luciferase Reporter Assay System (Promega). The relative value of the firefly luminescence to that of the *Renilla* luminescence ratio was calculated for each sample well.

### 2.16. Endothelial Cell Monolayer Permeability Assay

The permeability of HPMECs was evaluated on the basis of the paracellular permeability of fluorescein isothiocyanate (FITC)–dextran into the lower chamber. HPMECs were grown on 0.4 *μ*m Transwell inserts. After the indicated duration for each treatment, 0.5 mL of FITC–dextran (1 mg/mL) was added to the upper wells and 1 mL of medium was added to the bottom chamber. After incubation in the dark for one hour, 50 *μ*L of the medium in the bottom chamber was aspirated and measured using the Bio-Tek Synergy HT microplate reader at an excitation and emission wavelengths of 488 and 520 nm, respectively. The basal FITC–dextran permeability for unstimulated monolayers was set at 100%.

### 2.17. Western Blot Analysis

Cells and tissues in each treatment group were homogenized in RIPA lysis buffer with protease inhibitor to extract protein. The BCA Protein Assay Kit was used for the quantitation of total protein. Equal amounts of protein from each biological replicate were subjected to western blotting as previously described [14].

### 2.18. Quantitative Real-Time Polymerase Chain Reaction (qRT-PCR)

Total RNA was extracted from tissues and cells using RNAiso Plus (Takara Bio Inc., Kusatsu, Shiga, Japan) and quantified using a Nano Photometer N50 (Implen, München, Germany). Then, 1 *μ*g of total RNA was used to amplify the complementary DNA (cDNA) using a Prime Script RT Reagent Kit with gDNA Eraser (Takara Bio Inc.). This cDNA (2 *μ*L) was used for qPCR amplification in a 25 *μ*L reaction system containing 12.5 *μ*L of TB Green Premix Ex Taq II (Takara Bio Inc.). Each sample was analyzed in triplicate, and the mean Ct value was used in the final data analysis. Relative gene expression was normalized to *β*-actin as an internal control and determined using the comparative threshold cycle (Ct) method (2^^-*ΔΔ*Ct^).

### 2.19. miRNA Purification and Quantitation

Exosomal RNAs from serum and cell supernatants were isolated using the exoRNeasy Serum/Plasma Maxi Kit (QIAGEN, Hilden, Germany). Total RNA in the tissues was isolated using RNAiso for Small RNA (Takara Bio Inc.) following the manufacturer's protocol, and then 50 ng of exosomal RNA was reverse-transcribed into cDNA using a Mir-X miRNA First-Strand Synthesis Kit (Takara Bio Inc.). Quantification of miRNA by qPCR was performed using TB Green Premix Ex Taq II (Takara Bio Inc.). The miR-122 sequence is identical among vertebrates. Thus, the entire miR-122 sequence (UGGAGUGUGACAAUGGUGUUUG) was used as a specific 5′ primer according to the miRBase Sequence Database. Each sample was analyzed in triplicate, and the mean Ct value was used in the final data analysis. The Ct values of miRNA were normalized to U6 snRNA as an internal control. Relative miRNA levels were estimated by calculating 2^−*ΔΔ*CT^ values.

### 2.20. Immunofluorescence

Coverslips were coated with poly-L-lysine overnight at room temperature. Cells were rinsed with PBS, fixed in 4% paraformaldehyde for 10 minutes at room temperature, and permeabilized with PBS containing 0.25% Triton X-100. Cells were blocked in PBS-Tween (PBST) containing 10% serum from the species in which the secondary antibody was raised for 30 minutes and incubated with the indicated primary antibodies in PBST in a humidified chamber at 4°C overnight. Cells were then incubated with Alexa Fluor 488- or Alexa Fluor 594-labeled secondary antibodies (Abcam, Cambridge, MA, USA) for 1 hour at room temperature in the dark. Phalloidin-iFluor 594 (Abcam) was used to detect F-actin. After the slides were washed and sealed, the cells were imaged using inverted microscopy (TE2000-U; Nikon).

### 2.21. *In Vitro* Matrigel Tube Formation Assay and *In Vivo* Matrigel Plug Angiogenesis Assay

An *in vitro* angiogenesis assay was conducted to evaluate the formation of three-dimensional tubular structures by endothelial cells cultured on Matrigel (BD, Franklin Lakes, NJ, USA). HPMEC suspensions (1 × 10^5^ cells/mL) were seeded into *μ*-Slide angiogenesis plates (ibidi GmbH, Lochhamer Schlag, Germany), which were precoated with Matrigel. After the corresponding interventions, the cells were cultured at 37°C with 5% CO_2_ for 6 hours, and the development of tubular structures was examined using an inverted microscope. Tube formation was quantified by measuring the lengths of tubes from each well using ImageJ (Media Cybernetics, Atlanta, GA, USA). A Matrigel plug angiogenesis assay was conducted to detect the newly formed blood vessels in the transplanted gel plugs in mice. After mixing high concentration Matrigel (Corning Incorporated Life Sciences, Tewksbury, MA, USA) with an endothelial cell suspension subjected to particular treatments, the mixture was injected into mice subcutaneously. After 14 days of inoculation, the Matrigel plug was excised, fixed with formalin, and stained with endothelial cell marker the CD31(1 : 2,000, Cell Signaling Technology) to detect blood vessels via immunohistochemistry. CD31staining-positive endothelial markers indicated the presence of newly formed capillaries in the sectioned gel plugs.

### 2.22. Cell Viability Assays

Cell viability was evaluated using an ethynyldeoxyuridine (EdU) assay, which was performed to detect proliferating cells using the Click-iT EdU Alexa Fluor 594 Imaging Kit (Invitrogen, California, USA) according to the manufacturer's protocol. Bright red fluorescence was analyzed using fluorescence microscope (TE2000-U; Nikon). The EdU-positive cells were counted in three randomly selected fields of each slide. EdU-positive cells were counted in three randomly selected fields on each slide.

### 2.23. Cell Death Assays

Annexin V-FITC/propidium iodide (PI) staining (Invitrogen) was used to detect apoptosis. Briefly, after the indicated treatments, cells were collected and resuspended in 200 *μ*L of binding buffer containing 5 *μ*L of Annexin V-FITC. After a 10 minute incubation, cells were washed and resuspended in 190 *μ*L binding buffer with 10 *μ*L PI (20 *μ*g/mL); then, the stained cells were detected using flow cytometric analysis. Apoptotic DNA fragmentation was detected using a terminal deoxynucleotidyl transferase (TdT)-mediated dUTP nick end labeling (TUNEL) assay (Click-iT TUNEL Alexa Fluor 488 Imaging Assay; Invitrogen). Green fluorescence-labeled DNA strand breaks were monitored using a fluorescence microscope. Positive cells were counted in three randomly selected fields on each slide.

### 2.24. Detection of ROS and Superoxide Production

Cellular ROS was detected by JC-1 dye using flow cytometry; this dye reversibly changes its color from green to orange as mitochondria membrane potentials increase. Superoxide production in lung tissue was detected by dihydroethidium (DHE, Invitrogen) staining using fluorescence microscopy; DHE stains the oxidized cell as a bright fluorescent red. The positive cells were counted in three randomly selected fields of each slide.

### 2.25. Glutathione (GSH) Detection

Samples were prepared according to the manufacturer's protocol. Briefly, 50 *μ*L of standards or prepared samples was added to the well, and 25 *μ*L detection reagent was then added. After mixing, samples were incubated for 15 minutes at room temperature and measured at Ex/Em = 390/510 nm to calculate free GSH concentration.

### 2.26. Statistical Analysis

Data are presented as the mean ± standard deviation (S.D.) for continuous variables that were normally distributed. Data are representative of, at least, three independent experiments in triplicate samples. Unpaired Student's *t*-tests or the Mann–Whitney *U* tests were used to compare continuous variables that were normally or abnormally distributed between two independent groups. One-way ANOVA followed by Tukey's post hoc tests was used to compare abnormally distributed continuous variables among three or more independent groups. Statistical significance was set at *P* < 0.05 with 95% confidence intervals. Significant differences are shown by ∗*P* < 0.05, ∗∗*P* < 0.01, and ∗∗∗*P* < 0.001. All statistical analyses were conducted using GraphPad Prism 7.0 (GraphPad Software, Inc., La Jolla, CA, USA).

## 3. Results

### 3.1. HFD-Induced Obesity Protects against ARDS by Promoting Pulmonary Endothelial Barrier and Attenuating Pathological Fibroproliferation *In Vivo*

**Figure 3:**
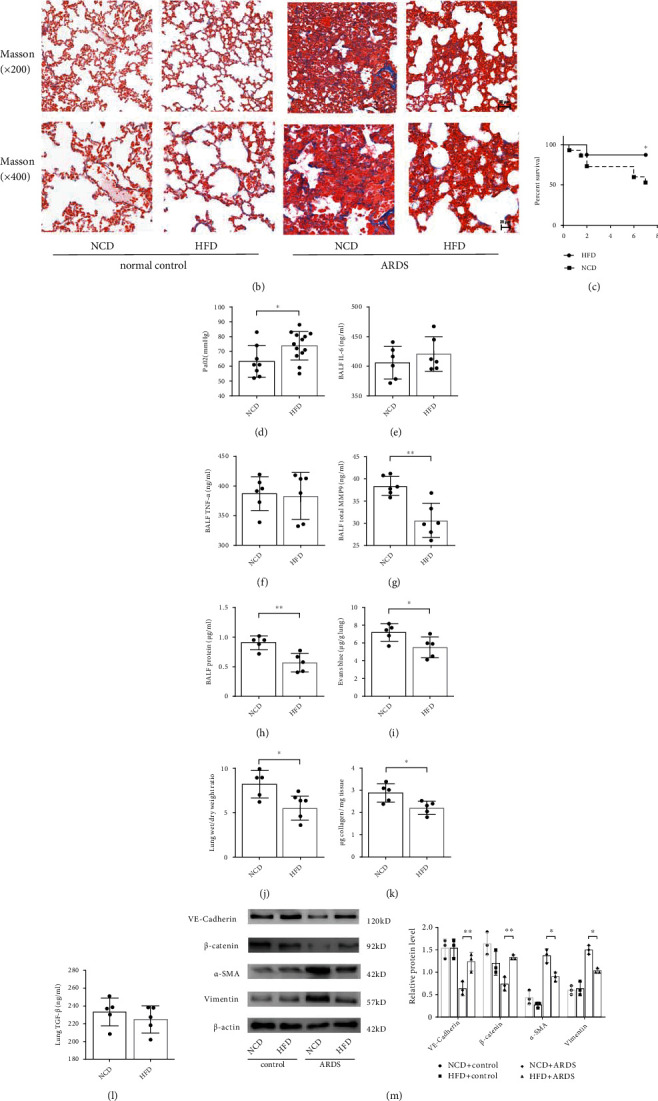
High-fat diet-induced obesity protects against ARDS by promoting pulmonary endothelial barrier and attenuating pathological fibroproliferation in mice. Histopathologic alterations of lung tissue by hematoxylin and eosin (H&E) staining (a) and Masson's trichrome staining (b) in the normal chow diet (NCD) induced lean control mice and high-fat diet (HFD) induced obese mice under normal and ARDS conditions (Scale bar = 50 *μ*m for magnification ×200 and scale bar = 20 *μ*m for magnification ×400). Representative images are shown from three replicated independent experiments. Comparison of mortality (c), PaO_2_ (d), the level of IL-6 (e), TNF-*α* (f), MMP9 (g), and protein (h) in bronchoalveolar lavage fluid (BALF), Evans blue-dyed albumin (EBDA) extravasation (i), wet/dry ratio (j), and collagen content (k) and TGF-*β* concentration in lung homogenate (l) between the NCD and HFD fed mice with ARDS. *n* = 16 mice per group (c). *n* = 8 mice for NCD group, and *n* = 13 mice for HFD group (d). *n* = 6 mice per group (e)–(g). *n* = 5 mice for the other group. (m) Western blot analysis of the protein expression of VE-cadherin, *β*-catenin, *α*-SMA, and Vimentin in the lung tissue. (n) Western blot analysis of the protein expression of TGF-*β* and TGF*β*R1 in the lung tissue. Relative abundances of protein bands were normalized to *β*-actin as shown in the bar graphs. Relative phosphorylation levels of protein are expressed normalized to the corresponding total protein. *n* = 3 mice per group analyzed in three replicated independent experiments. Data are presented as mean ± S.D. Significant differences are shown by ∗*P* < 0.05, ∗∗*P* < 0.01, and ∗∗∗*P* < 0.001.

Compared with age- and strain-matched NCD-fed controls, HFD-induced obese mice had a greater total body weight and exhibited adipocyte hypertrophy, as well as accumulation of intrahepatic fat and disordered metabolism, which confirmed the altered body composition (Figure S1). To investigate the effects of HFD-induced obesity on ARDS *in vivo*, lung histopathology, blood gas, inflammation, alveolocapillary permeability, and pulmonary fibroproliferative changes were examined comprehensively. Compared with lean control mice, lung injury and fibrosis were mitigated in obese mice with ARDS (Figures 3(a) and 3(b)), accompanied by a lower mortality (Figure 3(c)) and higher PaO_2_ (Figure 3(d)). However, no significant differences in the concentration of IL-6 and TNF-*α* in BALF (Figures 3(e) and 3(f)) were found between two groups, suggesting that the protective effects of obesity in ARDS may be mediated via inflammation-independent mechanisms. The pulmonary endothelial barrier is a crucial target in ARDS. Therefore, we further examined the effects of obesity on the pulmonary endothelial barrier homeostasis *in vivo*. We found that obesity attenuated exacerbations of capillary leakage, as manifested by decreases in BALF protein concentrations (Figure 3(h)), EBDA extravasation (Figure 3(i)), and W/D ratios (Figure 3(j)). Furthermore, consistent with the mitigation of pulmonary fibroproliferation, collagen levels in lung tissue and MMP9 concentrations in BALF were significantly lower in obese mice with ARDS (Figures 3(k) and 3(g)). These findings indicate a beneficial role of HFD-induced obesity on the pulmonary endothelial barrier and pathological fibroproliferation under the ARDS condition *in vivo*.

EndMT is known to be the key intersection of endothelial dysfunction and mesenchymal fibrosis. Thus, to investigate the molecular mechanisms underlying the favorable effects of obesity in ARDS, the expression of endothelial and mesenchymal markers was measured. We found that the expression of endothelial markers VE-cadherin and *β*-catenin in the lung tissue lysate of obese mice was significantly enhanced compared with that of lean control mice. Consistent with the mitigation of fibrosis, the expression of *α*-smooth muscle actin (*α*-SMA) and Vimentin was lower in the lungs of obese mice than that in the lungs of lean mice (Figure 3(m)). As a canonical profibrotic pathway, the TGF-*β* pathway is involved in the regulation of EndMT. Thus, we explored the alterations in TGF-*β* and TGF*β*-R1 expressions in lung tissue. We found no significant difference in TGF-*β* expression between the two groups, whereas TGF-*β*R1 expression was lower in obese mice with ARDS (Figures 3(l) and 3(n)). Collectively, HFD-induced obesity exerts beneficial effects in ARDS by reinforcing the pulmonary endothelial barrier and attenuating pathological fibroproliferation *in vivo*, suggesting an inhibition of the EndMT process.

### 3.2. Circulating Exosomes from Obese Mice Promote the Function and Integrity of the Pulmonary Endothelial Barrier in ARDS by Inhibiting EndMT

**Figure 4:**
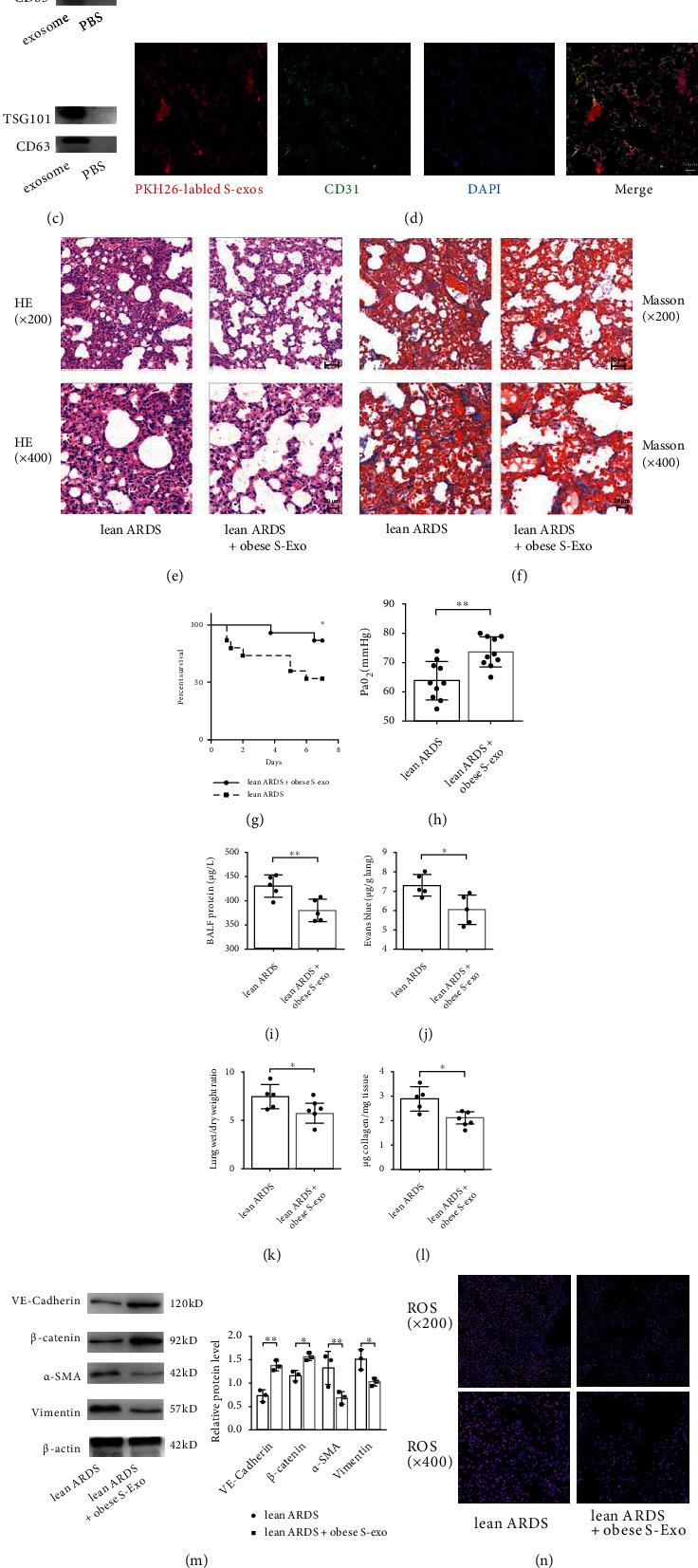
Obesity promotes pulmonary endothelial barrier and attenuates pathological fibroproliferation and oxidative stress *via* circulating exosomes. (a) Transmission electron microscope (TEM) analysis of serum exosomes from obese or lean control mice. Scale bar = 200 nm (left panel) and 100 nm (right panel). (b) Particle size and concentration of exosomes were detected by nanoparticle tracking analysis (NTA). (c) Exosome-specific marker TSG101 and extracellular vesicle-related protein marker CD63 were verified by western blot analysis. (d) PKH-26 dye labeled exosomes were intravenously administered to recipient mice. The efficient uptake of exosomes was confirmed by the appearance of red fluorescent PKH-26 dye in pulmonary capillaries, which were labeled with the endothelial marker CD31 (green fluorescence). Scale bar = 50 *μ*m. Histopathologic alterations of lung tissue by H&E (e) and Masson's trichrome staining (f) in lean mice with ARDS, which were pretreated with exosomes from obese mice serum (100 *μ*g/mL in a total volume of 300 *μ*L of PBS every week for three weeks). Representative images are shown from three replicated independent experiments. The mortality (g), PaO_2_ (h), BALF protein (i), EBDA extravasation (j), wet/dry ratio (k), and collagen content (l) in lean mice subjected to ARDS and those pretreated with exosomes from obese mice serum. *n* = 15 mice per group (g). *n* = 10 mice per group (h). *n* = 5 mice for the other group. (m) Western blot analysis of the protein expression of VE-cadherin, *β*-catenin, *α*-SMA, and Vimentin in the lung tissue. Relative abundances of protein bands were normalized to *β*-actin as shown in the bar graphs. *n* = 3 mice per group analyzed in three replicated independent experiments. (n) ROS production and (o) free GSH concentration in lung tissue.

Exosomes were harvested from the serum of mice, and the ultrastructure and size of these exosomes were observed using TEM and NTA, showing a typical cup-shaped morphology with an average diameter of approximately 100 nm (Figures 4(a) and 4(b)). The concentration of serum exosomes was significantly higher in obese mice than in control mice (2.10 ± 0.72 vs 8.90 ± 1.38 × 10^10^ Particles/mL, *P* = 0.002). Presence of the exosome-specific marker TSG101 and the extracellular vesicle-related protein marker CD63 was verified using western blot analysis (Figure 4(c)). To further unravel the role played by circulating exosomes in obesity-mediated attenuation of ARDS, lean mice were intravenously pretreated with exosomes from the serum of obese mice, and then subjected to ARDS for 7 days. Exosomes were labeled with PKH26 dye and intravenously administered to mice. The appearance of red fluorescent PKH26 dye in the lung tissue capillaries that were labeled with the endothelial marker CD31 confirmed the efficient uptake of exosomes (Figure 4(d)). To estimate endothelial barrier function *in vivo*, histopathologic changes, BALF protein concentrations, EBDA extravasation, and lung W/D ratios were measured. Pretreatment with exosomes from obese mice improved the survival (Figure 4(g)) and oxygenation (Figure 4(h)) of lean mice subjected to an ARDS insult. Exosomes from obese mice significantly mitigated pulmonary injury (Figure 4(e)) and hyperpermeability in lean mice with ARDS, as shown by decreases in BALF protein concentrations (Figure 4(i)), EBDA extravasation (Figure 3(j)), and W/D ratios (Figure 3(k)). Furthermore, exosomes from obese mice serum markedly attenuated pulmonary fibrosis (Figure 4(f)) and decreased the level of collagen in the lung tissue (Figure 4(l)) of lean mice with ARDS. Concomitantly, the expression of VE-cadherin and *β*-catenin in lung tissue was increased by the administration of exosomes from obese mice serum, whereas the expression of *α*-SMA and Vimentin was markedly decreased by this treatment (Figure 4(m)). Moreover, pretreatment with exosomes from obese mice limited oxidative stress and increased the free GSH concentration after ARDS insult (Figures 4(n) and 4(o)). These results indicate that circulating exosomes produced in the obese state mediate the protective effects of obesity in ARDS by inhibiting EndMT.

### 3.3. Obesity Enhances the Release of Circulating Exosomal miRNA-122-5p Triggered by ARDS *In Vivo*

**Figure 5:**
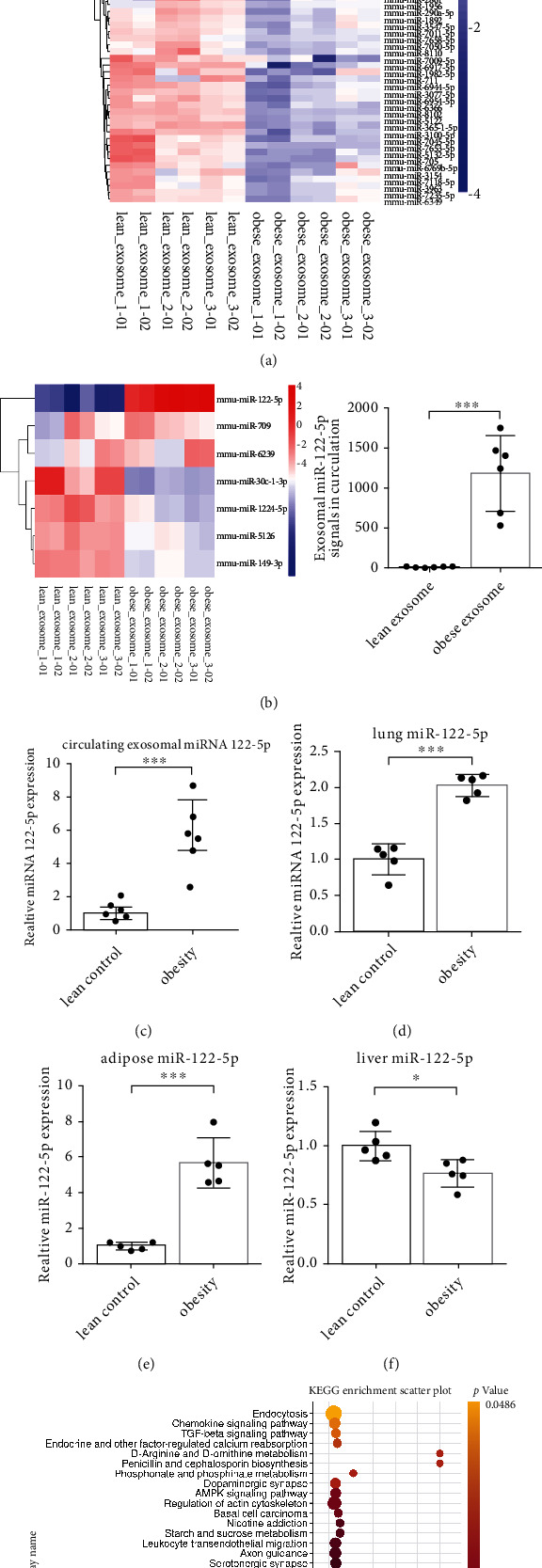
Obesity enhances the release of circulating exosomal miRNA-122-5p triggered by ARDS in mice. (a) Heatmap of circulating exosomal miRNAs in lean control and obese mice in the setting of ARDS showed the differences in the serum exosomal miRNA profiles. *n* = 9 mice per group, and each sample containing serum from 3 mice was analyzed in duplicate. (b) Among the miRNA transcripts with high signals (signal >500) that were significantly changed, miR-122-5p was the top-ranked different miRNA. qPCR identified that the levels of miR-122-5p were highly elevated in the circulation (c), lungs (d), and adipose tissue (e), whereas decreased in liver tissue (f) of obese mice under ARDS circumstance. (g) The Kyoto Encyclopedia of Genes and Genomes (KEGG) annotation system identified the TGF-*β* signaling pathway among the significant canonical pathways of miRNA-122-5p (*P* = 0.0016). *n* = 5 mice per group analyzed in three replicated independent experiments (c)–(e). The relative miRNA level was normalized to U6 snRNA as an internal control. Data are presented as mean ± S.D. Significant differences are shown by ∗*P* < 0.05, ∗∗*P* < 0.01, and ∗∗∗*P* < 0.001.

Adipose tissue constitutes a major source of the circulating exosomal miRNAs that can regulate biological processes in distant tissues [19]. Therefore, we profiled circulating exosomal miRNAs in lean control and obese mice in the context of ARDS, and then analyzed the differences in the circulating exosomal miRNA profiles. The miRNA array analysis revealed 147 and 160 significant differentially expressed exosomal miRNAs in the serum of lean and obese mice after ARDS insult, respectively (*P* < 0.05). Among these exosomal miRNAs, 70.07% (103/147) and 75.62% (121/160) were increased in lean and obese mice after the ARDS insult, respectively (Figure S2). Collectively, the acute stress from ARDS caused a significant release of exosomal miRNAs into circulation. Among 101 differentially expressed miRNAs between the lean and obese mice with ARDS (Figure 5(a)), miR-122-5p was the top-ranked differentially expressed miRNA with high abundance (signal >500) (Figure 5(b)). The up-regulation of exosomal miR-122-5p in the circulation and lung of obese mice was confirmed using qPCR (Figures 5(c) and 5(d)). It has been reported that miR-122-5p is predominantly expressed in the healthy liver tissue, whereas adipose tissue produces increased miR-122 into the circulation to compensate for the drop in hepatic production of miR-122 during obese-related disease [25]. We found highly elevated levels of miR-122-5pin in the adipose tissue of obese mice (Figure 5(e)) but decreased levels in their liver tissue (Figure 5(f)). KEGG annotation system identified the TGF-*β* signaling pathway among the significant canonical pathways of miRNA-122-5p (*P* = 0.0016; Figure 5). Collectively, these data indicate that adipose-derived circulating exosomal miRNA-122-5p may mediate the beneficial effects of obesity in ARDS in an indirect manner involving the TGF-*β* signaling pathway.

### 3.4. Adipose-Derived Exosomal miRNA-122-5p Mediates Obesity's Protective Effects on the Pulmonary Endothelial Barrier by Inhibiting EndMT and Oxidative Stress in ARDS *In Vivo* and *In Vitro*

MiR-122-5p has been reported to be secreted predominately by adipose tissue within exosomes in obesity-related diseases and involved in the regulation of fibroproliferation [25]. Thus, we hypothesized that adipose-derived miR-122-5p may be transferred to lung tissue through circulating exosomes thus exerting obesity's beneficial effects in ARDS.

**Figure 6:**
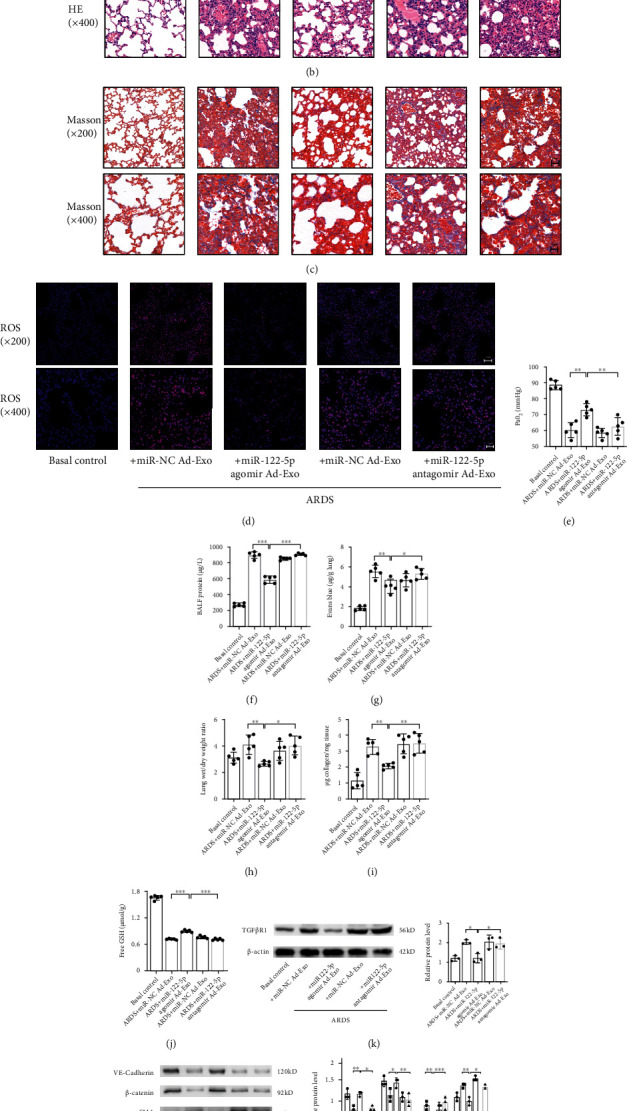
Adipose-derived exosomal miRNA-122-5p mediates obesity's protective effects on the pulmonary endothelial barrier and pathological fibroproliferation by inhibiting EndMT and oxidative stress in mice with ARDS. (a) The red fluorescence PKH-26 dye in the CD31-marked pulmonary capillaries confirmed the efficient uptake of exosome in mouse model of ARDS. Scale bar = 50 *μ*m. Histopathologic alterations by H&E (b) and Masson's trichrome staining (c), and ROS production (d) in the lung tissue of mice with ARDS, which were pretreated with adipose-derived exosomes (100 *μ*g/mL in a total volume of 300 *μ*L of PBS every week for three weeks) transfected with miR-122-5p agomir (10 nmol) or miR-122-5p antagomir (50 nmol), as well as their respective negative control (NC). Representative images are shown from three replicated independent experiments. *n* = 3 mice per group. The PaO_2_ (e), BALF protein (f), EBDA extravasation (g), wet/dry ratio (h), collagen content (i), and free GSH concentration (j) in mice with ARDS that were pretreated with adipose-derived exosomes, which were transfected with miR-122-5p agomir or miR-122-5p antagomir or respective NC. *n* = 5 mice per group in three replicated independent experiments. (k) Western blot analysis of the protein expression of TGF*β*R1 in the lung tissue. (l) Western blot analysis of the protein expression of VE-cadherin, *β*-catenin, *α*-SMA, and Vimentin in the lung tissue. Relative abundances of protein bands were normalized to *β*-actin as shown in the bar graphs. *n* = 3 mice per group analyzed in three replicated independent experiments. Data are presented as mean ± S.D. Significant differences are shown by ∗*P* < 0.05, ∗∗*P* < 0.01, and ∗∗∗*P* < 0.001.

First, miR-122-5p agomir or miR-122-5p antagomir was transfected into the adipose tissue (Figure S3A); these modified exosomes were purified and labeled with PKH26 dye and then delivered to the lung tissue of mice. The 5-FAM-labled miR-122-5p agomir or miR-122-5p antagomir was detected in the lung tissue (Figure S3B). The red fluorescence of PKH26 dye in CD31-marked pulmonary capillaries confirmed the efficient uptake of exosomes *in vivo* (Figure 6(a)). In the context of ARDS, exosomes derived from the adipose tissue of lean mice, which were pretreated with miR-122-5p agomir, significantly mitigated lung injury, fibrosis, and ROS accumulation (Figures 6(b) and 6(d)), improved oxygenation and the free GSH concentration (Figures 6(e) and 6(j)), attenuated hyperpermeability (Figures 6(f)–6(h)), and decreased the collagen levels and TGF*β*-R1 expression in the lung tissue (Figures 6(i) and 6(k)). Concomitantly, the expression of endothelial markers was increased, whereas the expression of mesenchymal markers was decreased (Figure 6(l)). Moreover, the Matrigel plug angiogenesis assay showed that exosomal miR-122-5p promoted angiogenesis, as evidenced by an increase in CD31-positive newly formed blood vessels (Figure S4). Contrastingly, administration of exosomes derived from adipose tissue that were pretreated with miR-122-5p antagomir reversed these beneficial effects. These data suggest a beneficial role of adipose-derived exosomal miRNA-122-5p on the pulmonary endothelial barrier and pathological fibroproliferation in mouse model of ARDS through inhibition of EndMT and oxidative stress.

**Figure 7:**
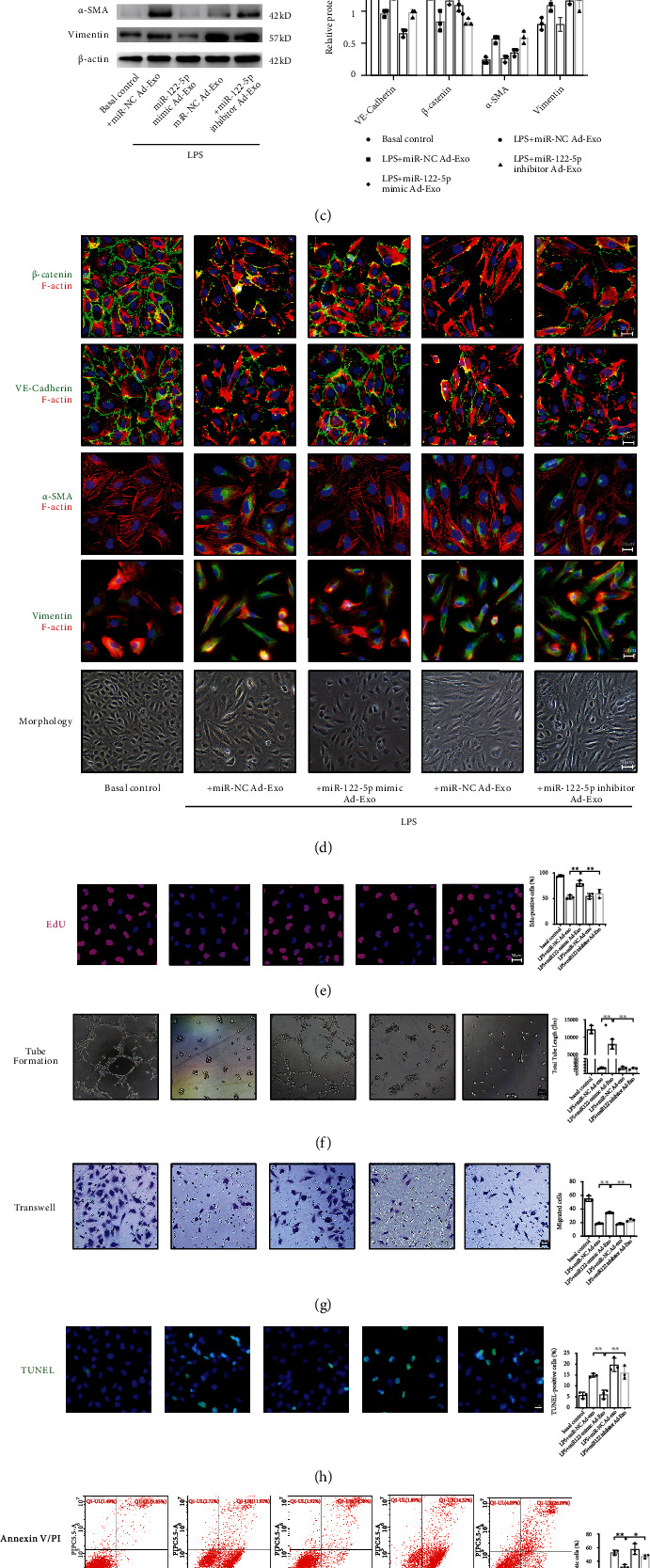
Adipocyte-derived exosomal miRNA-122-5p reinforces the pulmonary endothelial barrier by inhibiting EndMT and oxidative stress in HPMECs. (a) Adipocyte-derived exosomes were labeled with PKH-26 dye, and the appearance of red fluorescence in CD31-marked HPMECs confirmed that adipocyte-derived exosomes were taken up by HPMECs. Scale bar = 20 *μ*m. (b) Adipocytes transfected with 5-FAM-labeled miR-122-5p mimics were cocultured with HPMECs in Transwell plates. 5-FAM-labeled miR-122-5p mimics were delivered from adipocyte to HPMECs, as evidenced by the appearance of green fluorescence concomitant with a 5.19-fold increase in miR-122-5p abundance in HPMECs. (c) Western blot analysis of the protein expression of VE-cadherin, *β*-catenin, *α*-SMA, and Vimentin in HPMECs. Relative abundances of protein bands were normalized to *β*-actin, as shown in the bar graphs. (d) Immunofluorescence staining, phalloidin F-actin staining, and cell morphology of HPMECs showed a transition from the flattened endothelial phenotype to spindled mesenchymal-like phenotype, along with a decrease in VE-cadherin and *β*-catenin, and a slight increase in *α*-SMA and Vimentin under LPS insult conditions (100 ng/mL for 72 hours), which were attenuated by adipocyte-derived exosomes pretreated with miR-122-5p mimics. Scale bar = 20 *μ*m. EdU staining (e), tube formation (f), transwell (g), TUNEL staining (h), Annexin V/PI staining (i), and JC-1 staining (j) showed that adipocyte-derived exosomes that were transfected with miR-122-5p mimics increased EdU staining positive HPMECs, tube length, and cell mitigation, whereas attenuated apoptosis and oxidative stress under LPS stimulation. These effects were reversed by adipocyte-derived exosomes that were transfected with miR-122-5p inhibitor. Representative images are shown from three replicated independent experiments. *n* = 3 per group. Data are presented as mean ± S.D. Significant differences are shown by ∗*P* < 0.05, ∗∗*P* < 0.01, and ∗∗∗*P* < 0.001.

Next, adipocyte-derived exosomes were labeled with PKH26 dye, and the appearance of red fluorescence in CD31-marked HPMECs confirmed that these exosomes had been taken up by HPMECs (Figure 7(a)). To confirm that exosomal miRNAs could be taken up by HPMECs, adipocytes transfected with 5-FAM-labeled miR-122-5p mimics were cocultured with HPMECs in Transwell plates. HPMECs exhibited efficient uptake of adipocyte-secreted exosomal miRNA, as indicated by the appearance of green fluorescence concomitant with a 5.19-fold increase in miR-122-5p abundance in HPMECs (Figure 7(b)). Compared with mimic-NCs, exosomes pretreated with miR-122-5p mimics could protect HPMECs against LPS insult by suppressing EndMT, as evidenced by increased expression of VE-cadherin and *β*-catenin, decreased expression of *α*-SMA and Vimentin, and concurrent endothelial morphology maintenance and actin cytoskeleton stabilization, which are indicative of mitigation of EndMT *in vitro* (Figures 7(c) and 7(d)). Moreover, exosomal miR-122-5p mimics promoted the proliferation (Figure 7(e)), angiogenesis (Figure 7(f)), and migration (Figure 7(g)) of HPMECs while suppressing apoptosis and oxidative stress (Figures 7(h) and 7(j)) after LPS challenge. However, these alterations were partially reversed when HPMECs were pretreated with the exosomal miR-122-5p inhibitor. In summary, adipose-derived exosomal miR-122-5p can reinforce the pulmonary endothelial barrier and suppress fibrogenesis by inhibiting EndMT and oxidative stress in ARDS both *in vivo* and *in vitro.*

### 3.5. Adipose-Derived Exosomal miR-122-5p Promotes the Pulmonary Endothelial Barrier by Inhibiting EndMT and Oxidative Stress in ARDS *via* a TGF*β* Pathway *In Vitro* and *In Vivo*

We further explored the molecular mechanisms underlying the role of adipose-derived exosomal miRNA-122-5p through the canonical profibrotic factor TGF-*β*1. Since the sequence of miR-122 is identical across vertebrates, bioinformatics prediction analysis was performed to identify targets of miR-122-5p. However, we found no canonical binding site for miRNA-122-5p in the 3′-UTR of TGF-*β*1. The previous study found that miR-122-5p targets the TGF-*β*1 5′-UTR in humans in a non-“seed-region” base-pairing manner. Thus, the luciferase assay was performed to validate whether miRNA-122-5p binds directly to human TGF-*β*1. Specifically, luciferase reporter constructs with either a wild-type (WT) or mutant (MUT) 5′-UTR of human TGF-*β*1 were transfected into HPMECs along with either the miR-122-5p mimic or the miRNA-NC. The firefly luciferase activity of the reporter containing the 5′-UTR WT of human TGF*β*1 was decreased to 62% by miR-122-5p. In contrast, there was no significant reduction in the luciferase activity of the reporter containing the 5′-UTR MUT of TGF-*β*1, indicating that human TGF-*β*1 is a direct target of miR-122-5p through the noncanonical binding site in its 5′-UTR (Figure 1(a)).

To confirm that effects of exosomal miR-122-5p on the pulmonary endothelial barrier in ARDS were mediated through TGF-*β*1, HPMECs were treated with recombinant TGF-*β*1 protein or TGF-*β*1 neutralizing antibody (TGF-*β*1-Ab) after an LPS insult. TGF-*β*1 blocked the inhibitory effect of exosomal miR-122-5p on EndMT. However, TGF-*β*1-Ab overturned these effects (Figures 1(b) and 1(c)). In addition, the angiogenic and antioxidant capacities of exosomal miR-122-5p on HPMECs were abrogated by TGF*β*-1 treatment, but TGF-*β*1-Ab partially restored these capacities (Figures 1(d)–1(h)). These data implied that the inhibition of EndMT and promotion of angiogenesis by exosomal miR-122-5p were mainly mediated through TGF-*β* pathway. Moreover, when exosomal miR-122-5p mimic was delivered to the HPMECs, there was a reduction in TGF-*β*1 expression and the phosphorylation of TGF-*β*R1 and Smad2. Conversely, the miR-122-5p inhibitor reversed TGF-*β*1 expression levels, and the phosphorylation levels of TGF*β*-R1 and Smad2 recovered almost to the control levels in LPS-injured HPMECs *in vitro* (Figure 1(i)).

In mice, miR-122-5p was reported to target the CDS of mouse TGF-*β*R1. Similarly, luciferase assays proved that miR-122-5p targets mouse TGF-*β*R1 through a binding site in its CDS (Figure 2(a)). We found that administration of recombinant mouse TGF-*β*1 partially abolished the beneficial effects of adipose-derived exosomal miR-122-5p, whereas pretreatment with SB431542, an inhibitor of TGF-*β*R1 kinase, restored these favorable effects (Figure 2(b)–2(i)). Consistent with our prior *in vivo* results, we observed a reduction in TGF-*β*R1 expression, and decreased phosphorylation of TGF*β*-R1 and Smad2, whereas the expression of TGF-*β*1 remained unchanged (Figure 2(j)). Conversely, pretreatment with miR-122-5p antagomir impaired the beneficial effects of adipose-derived exosomes on ARDS *in vivo*, which is consistent with the enhanced expression of TGF-*β*R1 and phosphorylation of TGF-*β*R1 and Smad2 (Figure 2(j)). Taken together, these results demonstrate that adipose-derived exosomal miR-122-5p promotes protection of the pulmonary endothelial barrier and attenuates pathological fibroproliferation by inhibiting EndMT and oxidative stress through down-regulation of the TGF-*β*/TGF-*β*R1/Smad2 pathway.

## 4. Discussion

Obesity is an increasing global epidemic, and the prevalence of obesity is reported to be approximately 20% in the intensive care unit [12]. Although obesity increases the risk of mortality in the general population, it has been associated with improved survival among critically ill patients [10–12]. Similarly, a meta-analysis including 6,268 patients with ARDS revealed that obese patients with ARDS have lower mortality than those with a normal BMI [7, 8, 26]. This counterintuitive phenomenon is referred to as the “obesity paradox in ARDS”, and the underlying mechanism for this intriguing phenomenon has yet to be elucidated.

As an active endocrine organ, adipose tissue releases a variety of bioactive molecules known as adipokines that contribute to the pathogenesis of various diseases [27]. Researchers and our team have reported that adipokines are vital regulators of respiratory diseases, and a number of adipokines can protect against the development of lung injury through various mechanisms [16–18]. Recently, innovative evidence has indicated that adipose tissue constitutes a major source of circulating exosomal miRNAs, which act as novel forms of adipokines in the regulation of biological processes in distant tissues [19–22]; thus, a new mechanism of adipose–lung crosstalk in the obesity–ARDS paradox has been proposed. However, few studies have investigated this adipose–lung crosstalk. Our prior research on VILI demonstrated that HFD-induced obesity attenuates lung injury by promoting maintenance of the pulmonary endothelial barrier via adipose-derived exosomes [14], supporting the finding that HFD protected mice from VILI through mechanisms independent of neutrophil recruitment [13]. Another recent study demonstrated that obesity protects against cardiac ischemia/reperfusion injury via robustly releasing small extracellular vesicles from energetically stressed adipocytes [28]. As the role played by adipose tissue derived exosomal miRNAs in the pathogenesis of ARDS is less well documented, our present study aimed to further explore its underlying mechanisms.

Exosomes are robustly released under conditions of acute injurious, and accumulating evidence suggests the importance of involvement of exosomes in respiratory medicine [29]. Our current data shows that the acute stress from ARDS leads to a significant release of exosomal miRNAs into the circulation in both obese and lean control mice, as evidenced by more than 70% of exosomal miRNAs being increased after the ARDS insult in both lean and obese mice. Consistent with our findings, clinical trials support the beneficial role of leukocyte microparticles in improving the prognosis of ARDS [30]. However, we found that obesity changed the profile of circulating exosomal miRNAs in the context of ARDS, and the miR-122-5p transcript was the most significantly altered miRNA with a high abundance. Numerous miRNAs are closely associated with the differentiation of adipocytes and are dysregulated in obesity [31], among which circulating miR-122 levels are positively correlated with BMI in obesity and its related metabolic disorders, including insulin resistance and nonalcoholic fatty liver disease (NAFLD) [32–34]. Under normal physiological conditions, miR-122 is highly expressed in the liver and has tumor-suppressing, anti-inflammatory, and antifibrotic properties. However, the levels of miR-122 are significantly decreased in the liver but increased in the serum and adipose tissue of patients with NAFLD. The oscillation of miR-122 expression is proposed to be explained by the compensatory supply of miR-122 within exosomes released by adipose tissue [25, 35]. Our present study confirmed the excessive fat accumulation in fat and liver tissues with disordered glucose and lipid metabolism, which were accompanied by the up-regulation of exosomal miR-122-5p in the serum, adipose, and lung tissue of the obese mice following ARDS insult. MiR-122 has been reported to maintain a normal liver phenotype by suppressing liver inflammation and fibrosis in liver injury. As these two pathologic processes also play critical roles in endothelial barrier dysfunction in ARDS, they indicate a potential therapeutic strategy for ARDS. Thus, we speculate that adipose-derived exosomal miR-122-5p might function as a genetic adipokine that facilitates crosstalk between adipose tissue and lung tissue in ARDS.

The typical pathophysiology of ARDS involves interactions among multiple mechanisms, including increased microvascular permeability, exaggerated inflammation, mesenchymal fibrosis, and excessive ROS accumulation. Considering that lung tissue has a prominent place in the microvasculature, efficient restoration of the endothelial barrier is crucial for the treatment of ARDS. Generally, fibrosis arises from injured tissues and functions as a repair mechanism after injurious insults; however, progressive fibrosis and excessive deposition of extracellular matrix led to tissue remodeling and malfunction. Endothelial cells are important targets not only for hyperpermeability but also for fibrosis. Abnormal repair of the endothelial barrier following disruption contributes to diffuse pulmonary edema in the acute phase of ARDS and persistent fibrosis in the later phase, which can result in diminished quality of life, pulmonary dysfunction, and even death [36]. EndMT represents a key link in the complex interactions between endothelial barrier dysfunction and mesenchymal fibrosis under inflammatory conditions [4]. It also plays a vital role in vascular homeostasis and contributes to the development of pulmonary vascular remodeling, such as in pulmonary arterial hypertension and idiopathic pulmonary fibrosis [5, 6]. However, the role of EndMT in the pathogenesis of ARDS remains poorly understood. Through multiple experiments using a mouse model of ARDS, we demonstrated that HFD-induced obesity attenuates endothelial disruption and pathological fibroproliferation via adipose-derived circulating exosomes in ARDS, indicating a contribution of EndMT suppression in obesity's beneficial effects in ARDS.

EndMT is a phenotypic-switching process by which endothelial cells lose their intrinsic properties and acquire mesenchymal-like cellular traits. VE-cadherin is the main component of adherens junctions in endothelial cells and plays a vital role in the maintenance of interactions between adjacent cells through its extracellular domains, and its intracellular domains, which are further anchored to the actin cytoskeleton via *β*-catenin, thereby regulating permeability and fluid homeostasis [37]. Our cellular and molecular data clearly show that the suppression of EndMT by adipose-derived exosomal miR-122-5p mitigates pulmonary hyperpermeability, fibroproliferation, and oxidative stress in ARDS.

Current evidence suggests that EndMT is regulated by a complex orchestration of signaling pathways similar to the epithelial-to-mesenchymal transition, among which TGF-*β* signaling is the principal pathway [4, 6, 38]. Our bioinformatics analysis identified the TGF-*β* signaling pathway among the significant canonical pathways of miRNA-122-5p. MiRNAs are known as important modulators of EndMT. The level of miR-122 was reported to be associated with hepatic fibrosis, and reduced miR-122 levels in circulation are known to precede collagen accumulation in the liver [25, 34, 39]. Since a previous study reported differential TGF-*β* pathway miR-122 target sites in humans and mice [40], we performed a luciferase assay to verify this species-dependent miRNA targeting. Indeed, luciferase assay confirmed that human TGF-*β*1 is a target of miR-122-5p through a binding site in its 5′ − UTR, whereas miR-122-5p targets mouse TGF-*β*R1 through a binding site in its CDS. In addition, we found decreased expression of TGF-*β*1 in HPMECs and TGF-*β*R1 in mouse lungs due to overexpression of miR-122-5p. Regulation of the TGF-*β* pathway in vascular angiogenesis is mediated by TGF-*β*R1, which further interacts with the type II receptor and induces phosphorylation of specific members of the Smad family. In endothelial cells, TGF-*β*-induced Smad2 phosphorylation is much more stable than TGF-*β*-induced Smad5 phosphorylation; thus exerting inhibitory effects on endothelial proliferation and migration [41]. Consistent with these findings, we found that exosomal miR-122-5p-mediated promotion of the endothelial barrier was accompanied by down-regulation of the TGF-*β*1/TGF-*β*R1/Smad2 pathway and that inhibition of this pathway reversed the beneficial effects of exosomal miR-122-5p. However, we cannot exclude the possibility that other signaling pathways play a pivotal role in this process.

There are some limitations in our present study. Considering that the obesity induced by dietary high-fats can influence many biological functions, the integrated and counteractive effects of preexisting obesity on ARDS are rather intricate and confounding. In particular, metabolism and nutrition play significant roles in critical illness. Therefore, more efforts should be devoted to revealing the associations between glucose/lipid metabolism and endothelial barrier during the progression of ARDS [42]. Moreover, signal pathways mediated the protection of obesity via adipose-derived exosomes are never exclusive. Alternative pathways related to metabolism, inflammation, and oxidative stress will be investigated in our further studies. Given that lungs are highly vascularized and ARDS can lead to hypercoagulable conditions, exosomes derived from adipose tissue show promising application in cell-free therapies in critical illness including ARDS, due to their easy accessibility from a wide variety of sources and their low immunogenicity, which will facilitate the transport of genetic material [29]. Nevertheless, issues concerning the biosafety range, effective duration, and precise targeting of modified exosomes remain to be sufficiently addressed for optimized exosomal therapies in the future.

## 5. Conclusions

Our study found that HFD-induced obesity protects against ARDS in mice by reinforcing the endothelial barrier and attenuating pathological fibroproliferation and oxidative stress *via* adipose-derived circulating exosomes. Furthermore, we thoroughly demonstrated that adipose-derived exosomal miR-122-5p promotes protection of the pulmonary endothelial barrier by inhibiting EndMT and oxidative stress through down-regulation of the TGF-*β*1/TGF-*β*R1/Smad2 pathway, which may contribute to the protective nature and underlying mechanism of the obesity paradox in ARDS and indicate promising prospects for adipose-derived modified exosomes in cell-free therapies for ARDS.

## Data Availability

All data supporting the conclusions of this work were included within the article and supplementary material. The microarray data will be available and shared after the approval of authors for noncommercial use.

## Supplementary Materials

Supplementary Materials Figure S1 High-fat diet-induced obesity in mice. Comparison of altered body composition between mice fed with HFD and NCD from 5 weeks of age for 12 weeks. (A) Morphology of mice fed with HFD and NCD. Comparison of the body weight (B), fast blood glucose (C), total protein (D), albumin (E), total cholesterol (F), total triglycerides (G), LDL-C (H), HDL-C (I), ALT (J)and AST (K) between lean control mice and HFD-induced obese mice. (L) Pathological changes of subcutaneous adipose tissue, white adipose tissue, brown adipose tissue and liver tissue by H&E and Oil Red O staining (magnification of 200 and 400) in NCD-induced lean control mice and HFD-induced obese mice. Representative images are shown from three independent experiments. *n* = 20 per group (A–L). Data are presented as mean ± S.D. Significant differences are shown by ∗*P* < 0.05, ∗∗*P* < 0.01, ∗∗∗*P* < 0.001. Fig. S2 Acute stress from ARDS challenge causes significant release of exosomal miRNAs into circulation. Heatmap of circulating exosomal miRNAs showed that 147 and 160 significant differentially expressed exosomal miRNAs in the serum of lean (A) and obese (B) mice after an ARDS challenge, respectively (*P* < 0.05). Among these, 70.07% (103/147) and 75.62% (121/160) of exosomal miRNAs were increased in lean and obese mice after an ARDS challenge, respectively. *n* = 9 mice per group, and each sample containing serum from 3 mice was analyzed in duplicate. Figure S3 miR-122-5p agomir or miR-122-5p antagomir transfected into adipose tissue is transferred into lung tissue via exosomes. 5-carboxyfluorescein (5-FAM)-labeled miR-122-5p agomir (10 nmol) or miR-122-5p antagomir (50 nmol) was transfected into adipose tissue for three weeks (A). Modified adipose-derived exosomes (100 *μ*g/mL in a total volume of 300 *μ*L of PBS every week for three weeks) were intravenously injected into recipient mice and transferred to the lung tissue. The efficient transfection was monitored by the green fluorescence of 5-FAM (B). Representative images are shown from three replicated independent experiments. *n* = 3 mice per group analyzed in three replicated independent experiments. Fig. S4 Adipose-derived exosomal miR-122-5p promotes angiogenesis *in vivo*. *In vivo* Matrigel plug angiogenesis assay showed that adipose-derived exosomes transfected with miR-122-5p agomir (10 nmol) increased the formation of capillary-like tube structures (A) and CD31-positive newly formed blood vessels (B), whereas miR-122-5p antagomir (50 nmol) reversed this proangiogenic effect. Representative images are shown from three replicated independent experiments. *n* = 3 mice per group analyzed in three replicated independent experiments.
