# Supplementary figures and images for: Adipose-Derived Circulating Exosomes Promote Protection of the Pulmonary Endothelial Barrier by Inhibiting EndMT and Oxidative Stress through Down-Regulation of the TGF-β Pathway: A Potential Explanation for the Obesity Paradox in ARDS
Source: Oxid Med Cell Longev. 2022 May 5;2022:5475832. doi: 10.1155/2022/5475832 (PMC9098334; doi:10.1155/2022/5475832)

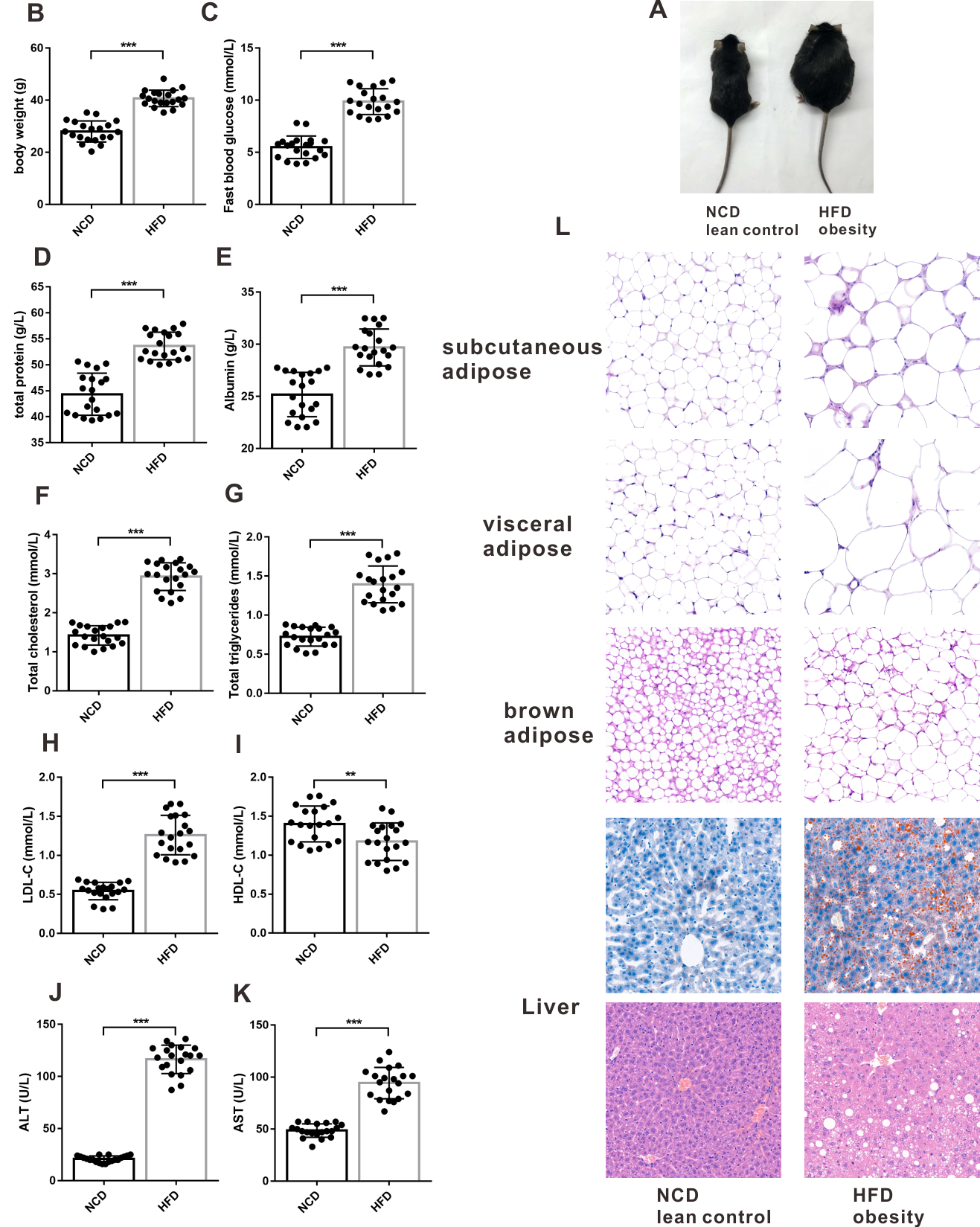

Supplement: Supplementary Materials — Figure S1 High-fat diet-induced obesity in mice. Comparison of altered body composition between mice fed with HFD and NCD from 5 weeks of age for 12 weeks. (A) Morphology of mice fed with HFD and NCD. Comparison of the body weight (B), fast blood glucose (C), total protein (D), albumin (E), total cholesterol (F), total triglycerides (G), LDL-C (H), HDL-C (I), ALT (J)and AST (K) between lean control mice and HFD-induced obese mice. (L) Pathological changes of subcutaneous adipose tissue, white adipose tissue, brown adipose tissue and liver tissue by H&E and Oil Red O staining (magnification of 200 and 400) in NCD-induced lean control mice and HFD-induced obese mice. Representative images are shown from three independent experiments. n = 20 per group (A–L). Data are presented as mean ± S.D. Significant differences are shown by ∗P < 0.05, ∗∗P < 0.01, ∗∗∗P < 0.001. Fig. S2 Acute stress from ARDS challenge causes significant release of exosomal miRNAs into circulation. Heatmap of circulating exosomal miRNAs showed that 147 and 160 significant differentially expressed exosomal miRNAs in the serum of lean (A) and obese (B) mice after an ARDS challenge, respectively (P < 0.05). Among these, 70.07% (103/147) and 75.62% (121/160) of exosomal miRNAs were increased in lean and obese mice after an ARDS challenge, respectively. n = 9 mice per group, and each sample containing serum from 3 mice was analyzed in duplicate. Figure S3 miR-122-5p agomir or miR-122-5p antagomir transfected into adipose tissue is transferred into lung tissue via exosomes. 5-carboxyfluorescein (5-FAM)-labeled miR-122-5p agomir (10 nmol) or miR-122-5p antagomir (50 nmol) was transfected into adipose tissue for three weeks (A). Modified adipose-derived exosomes (100 μg/mL in a total volume of 300 μL of PBS every week for three weeks) were intravenously injected into recipient mice and transferred to the lung tissue. The efficient transfection was monitored by the green fluorescence of 5-FAM (B). Representat [file 5475832.f1.zip › 5475832.f1/5475832.f1.jpg]

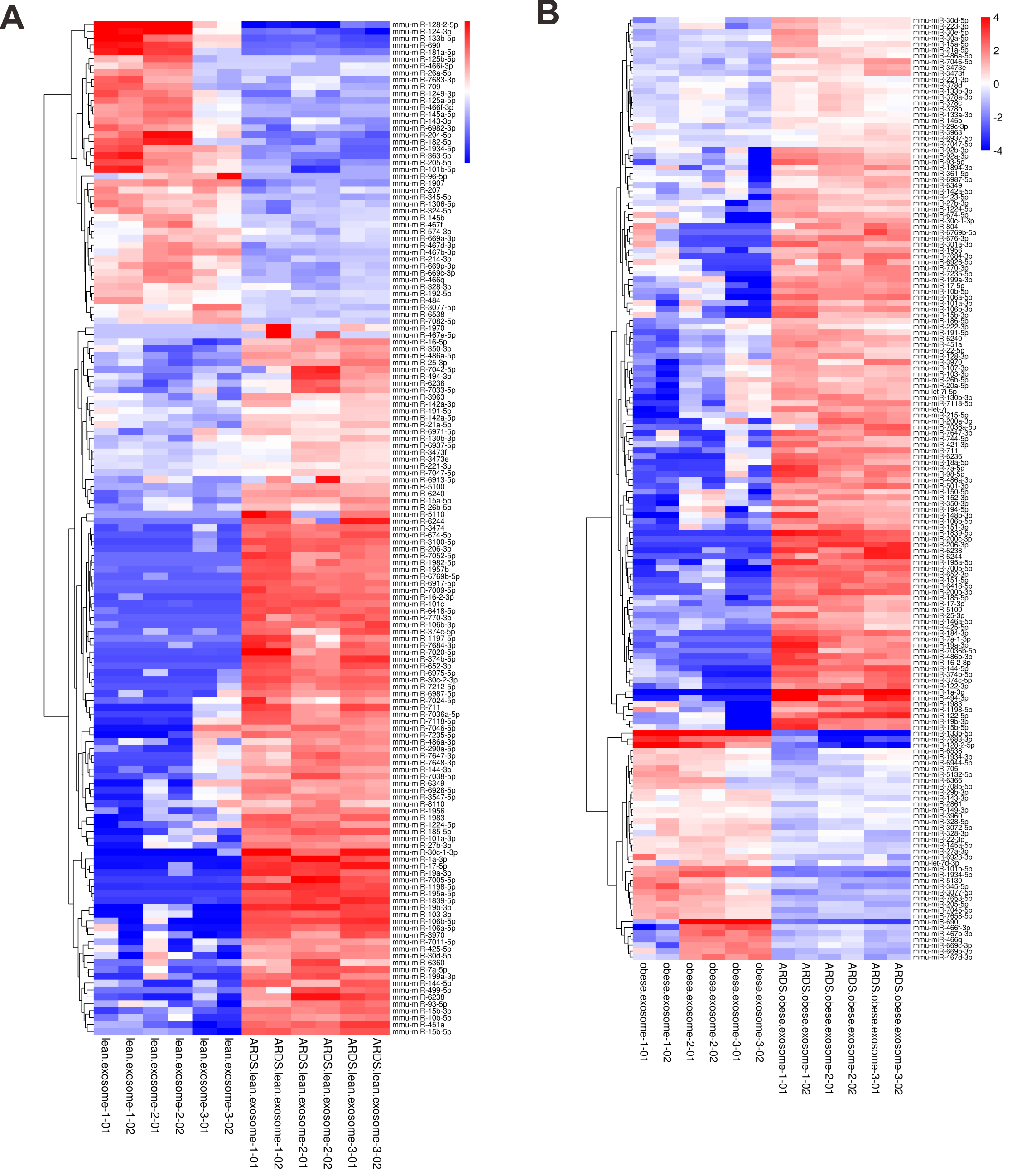

Supplement: Supplementary Materials — Figure S1 High-fat diet-induced obesity in mice. Comparison of altered body composition between mice fed with HFD and NCD from 5 weeks of age for 12 weeks. (A) Morphology of mice fed with HFD and NCD. Comparison of the body weight (B), fast blood glucose (C), total protein (D), albumin (E), total cholesterol (F), total triglycerides (G), LDL-C (H), HDL-C (I), ALT (J)and AST (K) between lean control mice and HFD-induced obese mice. (L) Pathological changes of subcutaneous adipose tissue, white adipose tissue, brown adipose tissue and liver tissue by H&E and Oil Red O staining (magnification of 200 and 400) in NCD-induced lean control mice and HFD-induced obese mice. Representative images are shown from three independent experiments. n = 20 per group (A–L). Data are presented as mean ± S.D. Significant differences are shown by ∗P < 0.05, ∗∗P < 0.01, ∗∗∗P < 0.001. Fig. S2 Acute stress from ARDS challenge causes significant release of exosomal miRNAs into circulation. Heatmap of circulating exosomal miRNAs showed that 147 and 160 significant differentially expressed exosomal miRNAs in the serum of lean (A) and obese (B) mice after an ARDS challenge, respectively (P < 0.05). Among these, 70.07% (103/147) and 75.62% (121/160) of exosomal miRNAs were increased in lean and obese mice after an ARDS challenge, respectively. n = 9 mice per group, and each sample containing serum from 3 mice was analyzed in duplicate. Figure S3 miR-122-5p agomir or miR-122-5p antagomir transfected into adipose tissue is transferred into lung tissue via exosomes. 5-carboxyfluorescein (5-FAM)-labeled miR-122-5p agomir (10 nmol) or miR-122-5p antagomir (50 nmol) was transfected into adipose tissue for three weeks (A). Modified adipose-derived exosomes (100 μg/mL in a total volume of 300 μL of PBS every week for three weeks) were intravenously injected into recipient mice and transferred to the lung tissue. The efficient transfection was monitored by the green fluorescence of 5-FAM (B). Representat [file 5475832.f1.zip › 5475832.f1/5475832.f2.jpg]

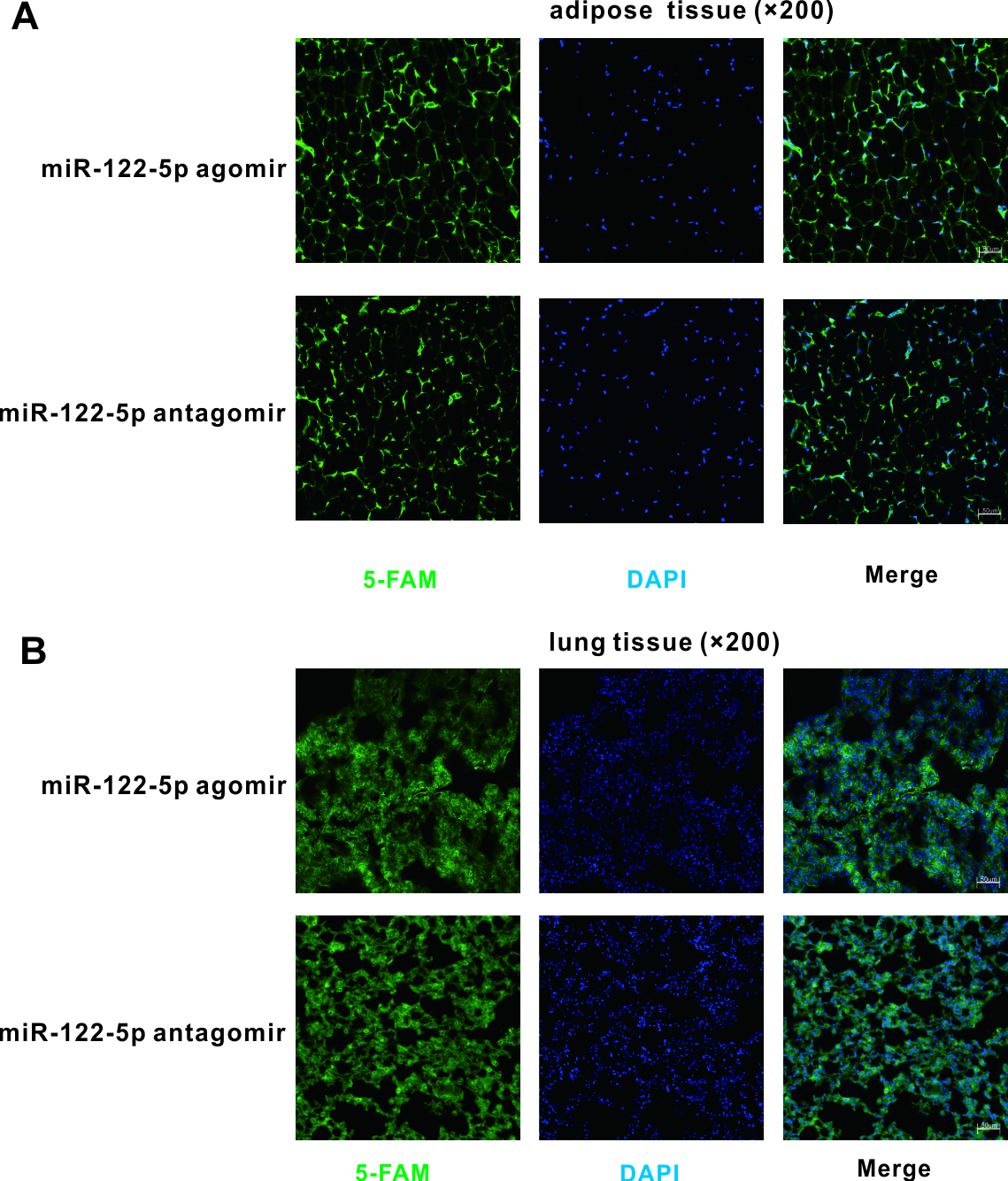

Supplement: Supplementary Materials — Figure S1 High-fat diet-induced obesity in mice. Comparison of altered body composition between mice fed with HFD and NCD from 5 weeks of age for 12 weeks. (A) Morphology of mice fed with HFD and NCD. Comparison of the body weight (B), fast blood glucose (C), total protein (D), albumin (E), total cholesterol (F), total triglycerides (G), LDL-C (H), HDL-C (I), ALT (J)and AST (K) between lean control mice and HFD-induced obese mice. (L) Pathological changes of subcutaneous adipose tissue, white adipose tissue, brown adipose tissue and liver tissue by H&E and Oil Red O staining (magnification of 200 and 400) in NCD-induced lean control mice and HFD-induced obese mice. Representative images are shown from three independent experiments. n = 20 per group (A–L). Data are presented as mean ± S.D. Significant differences are shown by ∗P < 0.05, ∗∗P < 0.01, ∗∗∗P < 0.001. Fig. S2 Acute stress from ARDS challenge causes significant release of exosomal miRNAs into circulation. Heatmap of circulating exosomal miRNAs showed that 147 and 160 significant differentially expressed exosomal miRNAs in the serum of lean (A) and obese (B) mice after an ARDS challenge, respectively (P < 0.05). Among these, 70.07% (103/147) and 75.62% (121/160) of exosomal miRNAs were increased in lean and obese mice after an ARDS challenge, respectively. n = 9 mice per group, and each sample containing serum from 3 mice was analyzed in duplicate. Figure S3 miR-122-5p agomir or miR-122-5p antagomir transfected into adipose tissue is transferred into lung tissue via exosomes. 5-carboxyfluorescein (5-FAM)-labeled miR-122-5p agomir (10 nmol) or miR-122-5p antagomir (50 nmol) was transfected into adipose tissue for three weeks (A). Modified adipose-derived exosomes (100 μg/mL in a total volume of 300 μL of PBS every week for three weeks) were intravenously injected into recipient mice and transferred to the lung tissue. The efficient transfection was monitored by the green fluorescence of 5-FAM (B). Representat [file 5475832.f1.zip › 5475832.f1/5475832.f3.jpg]

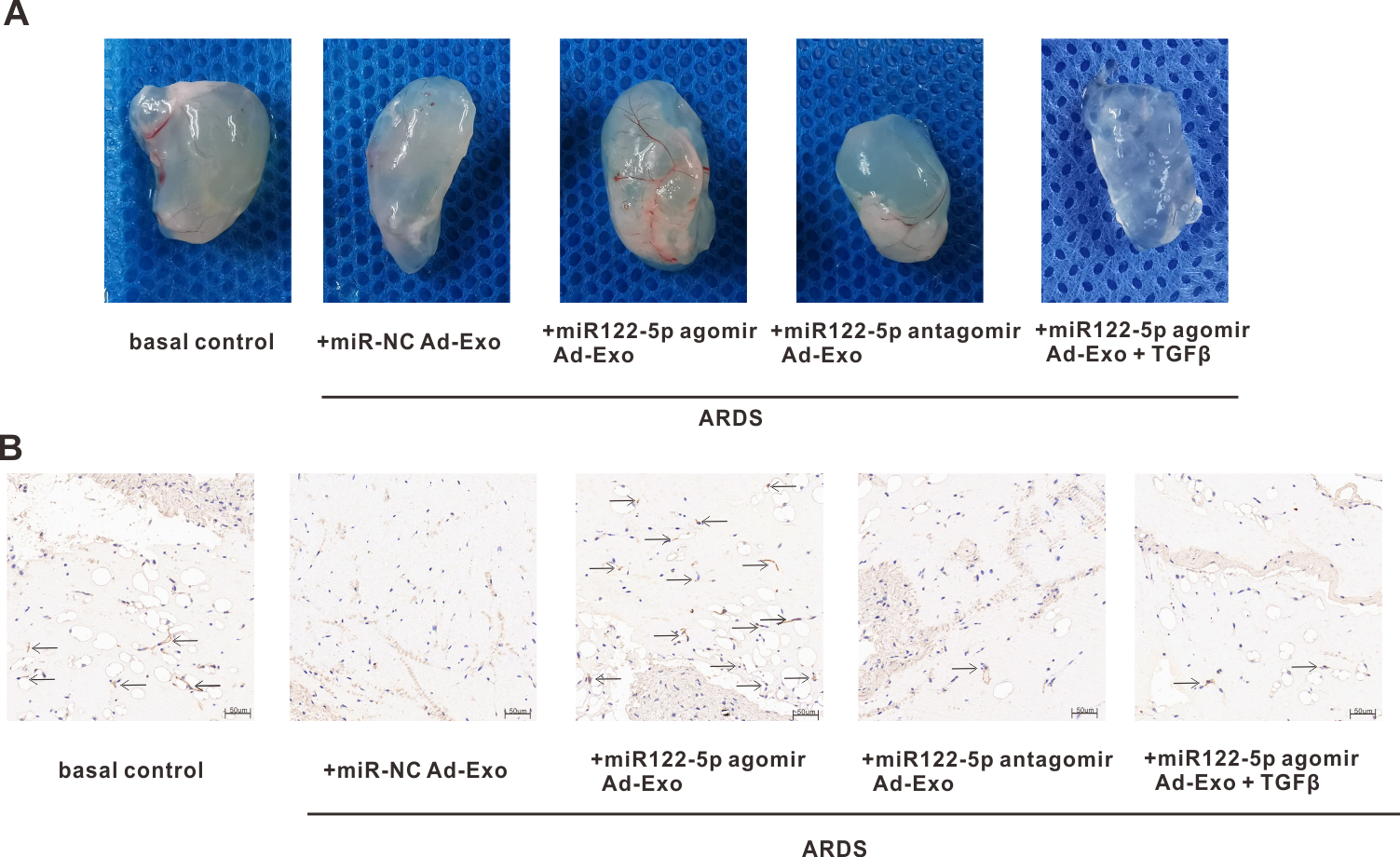

Supplement: Supplementary Materials — Figure S1 High-fat diet-induced obesity in mice. Comparison of altered body composition between mice fed with HFD and NCD from 5 weeks of age for 12 weeks. (A) Morphology of mice fed with HFD and NCD. Comparison of the body weight (B), fast blood glucose (C), total protein (D), albumin (E), total cholesterol (F), total triglycerides (G), LDL-C (H), HDL-C (I), ALT (J)and AST (K) between lean control mice and HFD-induced obese mice. (L) Pathological changes of subcutaneous adipose tissue, white adipose tissue, brown adipose tissue and liver tissue by H&E and Oil Red O staining (magnification of 200 and 400) in NCD-induced lean control mice and HFD-induced obese mice. Representative images are shown from three independent experiments. n = 20 per group (A–L). Data are presented as mean ± S.D. Significant differences are shown by ∗P < 0.05, ∗∗P < 0.01, ∗∗∗P < 0.001. Fig. S2 Acute stress from ARDS challenge causes significant release of exosomal miRNAs into circulation. Heatmap of circulating exosomal miRNAs showed that 147 and 160 significant differentially expressed exosomal miRNAs in the serum of lean (A) and obese (B) mice after an ARDS challenge, respectively (P < 0.05). Among these, 70.07% (103/147) and 75.62% (121/160) of exosomal miRNAs were increased in lean and obese mice after an ARDS challenge, respectively. n = 9 mice per group, and each sample containing serum from 3 mice was analyzed in duplicate. Figure S3 miR-122-5p agomir or miR-122-5p antagomir transfected into adipose tissue is transferred into lung tissue via exosomes. 5-carboxyfluorescein (5-FAM)-labeled miR-122-5p agomir (10 nmol) or miR-122-5p antagomir (50 nmol) was transfected into adipose tissue for three weeks (A). Modified adipose-derived exosomes (100 μg/mL in a total volume of 300 μL of PBS every week for three weeks) were intravenously injected into recipient mice and transferred to the lung tissue. The efficient transfection was monitored by the green fluorescence of 5-FAM (B). Representat [file 5475832.f1.zip › 5475832.f1/5475832.f4.jpg]
